# Supplementary material for: First unambiguous record of pneumaticity in the axial skeleton of alvarezsaurians (Theropoda: Coelurosauria)
Source: PLoS One. 2025 Apr 2;20(4):e0320121. doi: 10.1371/journal.pone.0320121 (PMC11964243; doi:10.1371/journal.pone.0320121)

PLOS ONE

First unambiguous record of pneumaticity in the axial skeleton of alvarezsaurians (Theropoda: Coelurosauria)

--Manuscript Draft--

**Manuscript Number:**	PONE-D-24-50106
**Article Type:**	Research Article
**Full Title:**	First unambiguous record of pneumaticity in the axial skeleton of alvarezsaurians (Theropoda: Coelurosauria)
**Short Title:**	First unambiguous record of axial skeleton pneumaticity in alvarezsaurians
**Corresponding Author:**	Michael Pittman<br>The Chinese University of Hong Kong<br>Shatin, Select a state... HONG KONG
**Keywords:**	Dinosauria; Theropoda; Alvareszauridae; Pneumaticity; Cretaceous; Patagonia
**Abstract:**	Bonapartenykus ultimus is an alvarezsaurid theropod from the Upper Cretaceous of Patagonia, Argentina. This species is represented by the holotype specimen and several referred specimens, which have particular bone features, such as the presence of pneumatic structures. Pneumaticity involves the invasion of the interior of the skeleton by means of air sac diverticula. Such invasion occurs from cortical openings (foramina) that communicate with internal air spaces. Despite the existence of previous studies on pneumatic structures in theropod specimens, there are no studies focusing on the family Alvarezsauridae. Thus, the first contribution focusing on the pneumatic features of an alvarezsaurid theropod is presented here. The specimens studied show that the axial skeleton of B. ultimus was invaded by pneumatic structures, reaching the middle section of the tail. Pneumaticity, among alvarezsaurids, does not suggest a linear evolutionary trajectory; but rather show to degree of random variability in the pneumatic features of the axial elements. Finally, the importance of computed tomographic images is also emphasized here, since a study on pneumaticity cannot be limited to the external anatomy of the specimens. Future studies will be essential to fully understand the extent of pneumatic invasion among alvarezsaurids to investigate its macroevolution aspects.
**Order of Authors:**	Guillermo J Windholz
	Jorge G. Meso
	Mathew J. Wedel
	Michael Pittman
**Additional Information:**	
**Question**	**Response**
**Financial Disclosure**<br><br>Enter a financial disclosure statement that describes the sources of funding for the work included in this submission. Review the submission guidelines for detailed requirements. View published research articles from *PLOS ONE* for specific examples.<br><br>This statement is required for submission and **will appear in the published article** if the submission is accepted. Please make	The author(s) received no specific funding for this work.

Powered by Editorial Manager® and ProduXion Manager® from Aries Systems Corporation

sure it is accurate.<br><br>**Funded studies**<br>Enter a statement with the following details:<br>• Initials of the authors who received each award<br>• Grant numbers awarded to each author<br>• The full name of each funder<br>• URL of each funder website<br>• Did the sponsors or funders play any role in the study design, data collection and analysis, decision to publish, or preparation of the manuscript?<br><br>Did you receive funding for this work?	
**Competing Interests**<br><br>Use the instructions below to enter a competing interest statement for this submission. On behalf of all authors, disclose any competing interests that could be perceived to bias this work—acknowledging all financial support and any other relevant financial or non-financial competing interests.<br><br>This statement is required for submission and **will appear in the published article** if the submission is accepted. Please make sure it is accurate and that any funding sources listed in your Funding Information later in the submission form are also declared in your Financial Disclosure statement.<br><br>View published research articles from *PLOS ONE* for specific examples.	The authors have declared that no competing interests exist.

**NO authors have competing interests**<br><br>Enter: *The authors have declared that no competing interests exist.*<br><br>**Authors with competing interests**<br><br>Enter competing interest details beginning with this statement:<br><br>*I have read the journal's policy and the authors of this manuscript have the following competing interests: [insert competing interests here]*<br><br>* typeset	
**Ethics Statement**<br><br>Enter an ethics statement for this submission. This statement is required if the study involved:<br><br>• Human participants<br>• Human specimens or tissue<br>• Vertebrate animals or cephalopods<br>• Vertebrate embryos or tissues<br>• Field research<br><br>Write "N/A" if the submission does not require an ethics statement.<br><br>General guidance is provided below. Consult the submission guidelines for detailed instructions. **Make sure that all information entered here is included in the Methods section of the manuscript.**	N/A. Specimens studied were previously accessioned specimens in institutional repositories.

Powered by Editorial Manager® and ProduXion Manager® from Aries Systems Corporation

**Format for specific study types**<br><br>**Human Subject Research (involving human participants and/or tissue)**<br>• Give the name of the institutional review board or ethics committee that approved the study<br>• Include the approval number and/or a statement indicating approval of this research<br>• Indicate the form of consent obtained (written/oral) or the reason that consent was not obtained (e.g. the data were analyzed anonymously)<br><br>**Animal Research (involving vertebrate animals, embryos or tissues)**<br>• Provide the name of the Institutional Animal Care and Use Committee (IACUC) or other relevant ethics board that reviewed the study protocol, and indicate whether they approved this research or granted a formal waiver of ethical approval<br>• Include an approval number if one was obtained<br>• If the study involved *non-human primates*, add *additional details* about animal welfare and steps taken to ameliorate suffering<br>• If anesthesia, euthanasia, or any kind of animal sacrifice is part of the study, include briefly which substances and/or methods were applied<br><br>**Field Research**<br><br>Include the following details if this study involves the collection of plant, animal, or other materials from a natural setting:<br>• Field permit number<br>• Name of the institution or relevant body that granted permission	
**Data Availability**<br><br>Authors are required to make all data underlying the findings described fully available, without restriction, and from the time of publication. PLOS allows rare exceptions to address legal and ethical concerns. See the PLOS Data Policy and FAQ for detailed information.	Yes - all data are fully available without restriction

A Data Availability Statement describing where the data can be found is required at submission. Your answers to this question constitute the Data Availability Statement and **will be published in the article**, if accepted.<br><br>**Important:** Stating 'data available on request from the author' is not sufficient. If your data are only available upon request, select 'No' for the first question and explain your exceptional situation in the text box.<br><br>Do the authors confirm that all data underlying the findings described in their manuscript are fully available without restriction?	
**Describe where the data may be found in full sentences. If you are copying our sample text, replace any instances of XXX with the appropriate details.**<br><br>• If the data are **held or will be held in a public repository**, include URLs, accession numbers or DOIs. If this information will only be available after acceptance, indicate this by ticking the box below. For example: *All XXX files are available from the XXX database (accession number(s) XXX, XXX.).*<br>• If the data are all contained **within the manuscript and/or Supporting Information files**, enter the following: *All relevant data are within the manuscript and its Supporting Information files.*<br>• If neither of these applies but you are able to provide **details of access elsewhere**, with or without limitations, please do so. For example:<br><br>*Data cannot be shared publicly because of [XXX]. Data are available from the XXX Institutional Data Access / Ethics Committee (contact via XXX) for researchers who meet the criteria for access to confidential data.*<br><br>*The data underlying the results presented in the study are available from (include the name of the third party*	All relevant data are within the manuscript and its Supporting Information files.

and contact information or URL).<br>• This text is appropriate if the data are owned by a third party and authors do not have permission to share the data.<br><br>* typeset	
Additional data availability information:	

Powered by Editorial Manager® and ProduXion Manager® from Aries Systems Corporation

Manuscript

Click here to access/download;Manuscript;Windholz et al. main text_20241103.docx

First unambiguous record of pneumaticity in the axial skeleton of alvarezsaurians (Theropoda: Coelurosauria)

G.J. Windholz*,[1], J.G. Meso[1], M.J. Wedel[2], M. Pittman*,[3]

[1]CONICET- Instituto de Investigación en Paleobiología y Geología, Sede Alto Valle-Valle Medio Universidad Nacional de Río Negro, Av. Roca 1242, Gral. Roca, Río Negro, Argentina. gwindholz@unrn.edu.ar jgmeso@unrn.edu.ar

[2]College of Osteopathic Medicine of the Pacific and College of Podiatric Medicine, Western University of Health Sciences, Pomona, California, 91711, USA. mathew.wedel@gmail.com

[3]School of Life Sciences, The Chinese University of Hong Kong, Hong Kong SAR, China. mpittman@cuhk.edu.hk

*corresponding authors

Abstract

Bonapartenykus ultimus is an alvarezsaurid theropod from the Upper Cretaceous of Patagonia, Argentina. This species is represented by the holotype specimen and several referred specimens, which have particular bone features, such as the presence of pneumatic structures. Pneumaticity involves the invasion of the interior of the skeleton by means of air sac diverticula. Such invasion occurs from cortical openings (foramina) that communicate with internal air spaces. Despite the existence of previous studies on pneumatic structures in theropod specimens, there are no studies focusing on the family Alvarezsauridae. Thus, the first contribution focusing on the pneumatic features of an alvarezsaurid theropod is presented here. The specimens studied show that the axial skeleton of *B. ultimus* was invaded by pneumatic structures, reaching the middle section of the tail. Pneumaticity, among alvarezsaurids, does not suggest a linear evolutionary trajectory; but rather show to degree of random variability in the pneumatic features of the axial elements. Finally, the importance of computed tomographic images is also emphasized here, since a study on pneumaticity cannot be limited to the external anatomy of the specimens. Future studies will be essential to fully understand the extent of pneumatic invasion among alvarezsaurids to investigate its macroevolution aspects.

Key words

Dinosauria, Theropoda, Alvareszauridae, Pneumaticity, Cretaceous, Patagonia

Introduction

Alvarezsauridae are a group of early diverging maniraptoran theropod dinosaurs, erected by Bonaparte [1]. The fossil records of this dinosaur family come from the Upper Cretaceous of South America, North America, Europe, and Asia [2–11]. Notably, the

diversity of this clade in South America is limited to Argentina, where five species described to date: *Alnashetri cerropoliciensis* from Candeleros Formation, Cenomanian [7, 12]; *Patagonykus puertai* from Portezuelo Formation, Coniacian [3, 13]; *Alvarezsaurus calvoi* and *Achillesaurus manazzonei* from Bajo de la Carpa Formation, Coniacian-Santonian [1, 4]; and *Bonapartenykus ultimus* from Allen Formation, middle Campanian–lower Maastrichtian [6].

Among the Argentinian forms has been identified an endemic subclade Patagonykinae currently including *Patagonykus puertai* and *Bonapartenykus ultimus* [6]. This subfamily showed a tendency toward larger body sizes, relative to other alvarezsaurians, with body length estimated to range from ~3 to ~3.3 meters [14]. Singularly, *Bonapartenykus ultimus* comes from the "Salitral Ojo de Agua" locality, Rio Negro Province (Patagonia Argentina) and it is the only known species of the genus. The species is represented by holotype specimen published by Agnolín et al. [6], along with several referred specimens published by Salgado et al. [15] and Meso et al. [16]. These specimens exhibit a particular bone anatomy, such as structures that would indicate the presence of postcranial pneumaticity.

Pneumaticity is a widely distributed feature among archosaurs, including pterosaurs and saurischian dinosaurs [17–24]. However, birds are the only living archosaurs with postcranial pneumaticity in their skeleton, since that crocodylians lack it [23]. The pneumatic system denotes the invasion of bone by air sac diverticula, which may manifest through observable external (such as fossae, foramina and laminae) and internal skeletal features (e.g., procamerate, camerate, and camellate) internal pneumatization pattern. Numerous studies have explored the pneumatic system of dinosaurs [e.g., 20, 21, 25–28]. Sauropods have been the main focus of attention [e.g., 19–21, 29, 30], perhaps due to the phenomenon of gigantism, but there have also been

studies investigating pneumaticity in non-avian theropods [e.i. 22, 23, 28, 31, 32, 34, 35]. Nevertheless, to date, no studies have focused on the pneumaticity of the family Alvarezsauridae. This study aims to fill that gap by characterizing the pneumatic structures in the axial skeleton of *Bonapartenykus* based on first-hand study of external anatomy, as well as both external and internal anatomy from computed tomography scans. We also compared the observed pneumatic pattern with other theropods, focusing on alvarezsaurians.

Materials and methods

The materials studied here come from the middle Campanian-lower Maastrichtian (Upper Cretaceous) Allen Formation of Río Negro Province, Argentina. The holotype specimen of *Bonapartenykus* (MPCA 1290) is housed in the MPCA "Museo Provincial Carlos Ameghino", Cipolletti City; while referred specimens (MPCN-PV 738) are housed in the MPCN "Museo Patagonico de Ciencias Naturales", located in General Roca City; both in Río Negro Province, Argentina.

The computed tomographic images (CTs) were obtained from eleven vertebral elements: a mid-cervical vertebra (MPCN-Pv 738.28), two posterior cervical vertebrae (MPCN-Pv 738.26 and 27), two cervico-dorsal vertebrae (MPCN-Pv 738.15 and 16), a sacral vertebra (MPCN-Pv 738.14), the first caudal vertebra (MPCN-Pv 738.10), an anterior caudal vertebrae (MPCN-Pv 738.29), two mid-caudal (MPCN-Pv 738.8 and 30) and a posterior caudal vertebra (MPCN-Pv 738.11). The scans were performed at "Sanatorio Juan XXIII" hospital in General Roca, Río Negro Province, Argentina. The remaining elements were examined first-hand for external anatomical features and to a limited extent some internal anatomy accessible through natural fractures in the fossils.

The descriptions follow the vertebral laminae and fossae nomenclature proposed by Wilson [36, 37] and Wilson et al. [38] as well as the terminology for pneumatic structures of Britt [17], Wedel [20] and O'Connor [22].

Results

Two anterior cervical vertebrae assigned to cf. *Bonapartenykus ultimus* (MPCN-PV 738) are preserved, a vertebral centrum (MPCN-Pv 738.32) and an incomplete neural arch (MPCN-Pv 738.47). The external surface of anterior cervical element (MPCN-Pv 738.32) lacks foramina. However, it presents a deep depression in its ventral region. This centrum is broken in its dorsal region, thus exposing a wide internal cavity surrounded by a reticule of interconnected trabeculae (Fig. 1A). Likewise, the neural arch (MPCN-Pv 738.47) bears poor developed laminae and shallow neural fossae (Fig. 1B). This vertebra has a pair of foramina on the inner surface of centrodiapophyseal fossa (**cdf**).

Figure 1. Anterior cervical vertebrae assigned to cf. *Bonapartenykus ultimus*. A, MPCN-Pv 738.32 in dorsal view; A_1, interpretive drawing showing internal air spaces; B, MPCN-Pv 738.47 in lateral view; B_1, detail of foramina. Abbreviations: **ic**, internal cavity; **ns**, neural spine; **prz**, prezygapophysis.

The mid-cervical region of cf. *Bonapartenykus ultimus* is represented by the vertebral element (MPCN-Pv 738.28). This preserved the neural arch with a rough appearance in its ventral surface indicating an open neurocentral suture (i.e., site of attachment with the centrum). This vertebral element presents a pattern of poorly developed neural laminae,

accompanied by wide lateral fossae (i.e., **cdf**, **prcdf**, **pocdf** and **sdf**) (Fig. 2A). Additionally, natural fractures in the preserved transverse processes and base of neural spine reveal a three-dimensional network of trabeculae, with small interconnected air spaces. Particularly, the inner surface of the prezygapophyseal centrodiapophyseal fossa (**prcdf**) and spinodiapophyseal fossa (**sdf**) of MPCN-Pv 738.28 bears foramina (Fig. 2A). Although externally mid-cervical neural fossae are shallow (Fig. 2B), CT scan images show a network of internally interconnected air spaces (Fig. 2B).

Figure 2. Mid-cervical vertebrae MPCN-Pv 738.28 assigned to cf. *Bonapartenykus ultimus*. A, MPCN-Pv 738.28 in lateral view; A_1, close ups showing foramina on **sdf** and A_2, on **prcdf**; B, three-dimensional reconstruction of MPCN-Pv 738.28 in lateral view; B_1, transverse section of MPCN-Pv 738.28 at mid-length, note the internal air spaces. Abbreviations: **cdf,** centrodiapophyseal fossa; **nc**, neural canal; **ns**, neural spine; **dp**, diapophysis; **prcdf**, prezygapophyseal centrodiapophyseal fossa; **prz**, prezygapophysis; **pocdf**, postzygapophyseal centrodiapophyseal fossa; **sdf,** spinodiapophyseal fossa.

The posterior cervical vertebrae assigned to cf. *Bonapartenykus ultimus* (MPCN-Pv 738.26 and 27) preserve only the neural arches. The pattern of neural laminae and fossae is considerably more complex compared to mid-cervical vertebrae (Fig. 3A and B). Lateral fossae (i.e., **cdf**, **prcdf**, **pocdf** and **sdf**) are deep as they almost reach the axial plane of the element. The posterior cervical vertebra (MPCN-Pv 738.27) has a deep spinopostzygapophyseal fossa (**spof**), with elliptical outline (Fig. 3C). This fossa reaches almost to the roof of the neural canal, invading almost half-height of the neural arch. The **spof** is not contrastable in vertebra (MPCN-Pv 738.26), since the posterior surface of the

neural spine is completely eroded. The inner surfaces of prezygapophyseal centrodiapophyseal fossae (**prcdf**) in posterior cervical vertebrae have a pair of foramina in MPCN-Pv 738.26 and a simple foramen in MPCN-Pv 738.27. Additionally, both posterior cervical vertebrae have a poorly developed lamina on the inner surface of the spinodiapophyseal fossae (**sdf**) and have a foramen on each side of that lamina; the only exception is the right **sdf** of MPCN-Pv 738.26, which is simple and has only one foramen inside it. Interesting, CTimages also show the interior of the neural arch with interconnected air spaces, including some small cameras in the neural spine (Fig. 3D and E).

Figure 3. Posterior cervical vertebrae assigned to cf. *Bonapartenykus ultimus*. A, MPCN-Pv 738.26 in lateral view, and detail of foramina (A_1); B and C, MPCN-Pv 738.27 in lateral (B), detail of foramina (B_1), and posterior views (C); D, three-dimensional reconstruction of MPCN-Pv 738.26 in lateral view, transverse (D_1), and frontal (D_2) sections; E, three-dimensional reconstruction of MPCN-Pv 738.27 in lateral view, transverse (E_1), and frontal (E_2) sections. Abbreviations: **cdf,** centrodiapophyseal fossa; **dp**, diapophysis; **nc**, neural canal; **ns**, neural spine; **prcdf**, prezygapophyseal centrodiapophyseal fossa; **pocdf**, postzygapophyseal centrodiapophyseal fossa; **sdf** spinodiapophyseal fossa; **spof**, spinopostzygapophyseal fossa.

The cervico-dorsal transition of cf. *Bonapartenykus ultimus* is represented by two incomplete vertebrae, which both MPCN-Pv 738.15 and 16 lacking the centrum, and an isolated fragment of postzygapophysis MPCN-Pv 738.48 and neural spine MPCN-Pv 738.49. Their neural fossae (**prsdf**, **posdf**, **sprf** and **spof**) are wide and deep (Fig. 4A-D),

especially the postzygapophyseal spinodiapophyseal fossa (**posdf**) that invades almost to the axial plane of the neural arch (Fig. 4E and F). The cervico-dorsal transition is characterized by the presence of deep spinoprezygapophyseal (**sprf**) and spinopostzygapophyseal (**spof**) fossae, of elliptical contour. This anatomical area is also represented by a fragment of isolated postzygapophysis (MPCN-Pv 738.48) and neural spine (MPCN-Pv 738.49), whose fractures show large internal cameras. The specimen (MPCN-Pv 738.15) has two foramina, separated by a thin bony layer on the interior surface of prezygapophyseal spinodiapophyseal fossa (**prsdf**). Ct images show that the interior of the neural spine of MPCN-Pv 738.15 and 16 has abundant interconnected air spaces (Fig. 4E and F).

Figure 4. Cervico-dorsal vertebrae assigned to cf. *Bonapartenykus ultimus*. A, MPCN-Pv 738.15 in lateral view, and detail of two foramina (A_1); B, C, and D, MPCN-Pv 738.16 in anterior (B), posterior (C), and lateral views (C); E, three-dimensional reconstruction of MPCN-Pv 738.15 in anterior view, frontal (E_1), and parasagittal (E_2) sections; F, three-dimensional reconstruction of MPCN-Pv 738.16 in lateral view and frontal section (F_1). Abbreviations: **ns**, neural spine; **posdf,** postzygapophyseal spinodiapophyseal fossa; **prsdf**, prezygapophyseal spinodiapophyseal fossa; **spof**, spinopostzygapophyseal fossa; **sprf**, spinoprezygapophyseal fossa.

The mid-dorsal vertebra (MPCA 1290, holotype specimen) was almost completely preserved and has been described in detail by Agnolín et al. [6] from an anatomical perspective. However, it is interesting to note some features that could be linked to the presence/absence of pneumaticity of the only vertebral element preserved in

the holotype specimen of *Bonapartenykus ultimus*. For example, the centrum lacks pleurocoels, although it bears a foramen on its lateral cortical surface. The natural fractures in the centrum would not appear to show signs of internal pneumaticity, such as camellas or cameras, although the preservation in that area is poor. The neural arch, in turn, presents well-developed lamellae that delimit wide and deep **pacdf**, **pocdf** and **sdf**; and a much reduced **prcdf** (Fig. 5A-B). Also, neural arch has a deep **spof** (Fig. 5C).

Figure 5. Mid-dorsal vertebrae of holotype specimen of *Bonapartenykus ultimus* (MPCA 1290). A, vertebra in lateral view and detail of foramina (A_1); B, three-dimensional reconstruction in lateral view; C, vertebral element in posterior view; D three-dimensional reconstruction in posterior view. Abbreviations: **nc**, neural canal; **pacdf**, parapophyseal centrodiapophyseal fossa; **pocdf**, postzygapophyseal centrodiapophyseal fossa; **prcdf**, prezygapophyseal centrodiapophyseal fossa; **sdf** spinodiapophyseal fossa; **spof**, spinopostzygapophyseal fossa.

The sacral region of cf. *Bonapartenykus ultimus* is represented by two anterior sacral vertebrae (MPCN-Pv 738.14) and the last sacral vertebra (MPCN-Pv 738.31). The poor state of preservation of those elements makes it difficult to observe certain features; however the natural fractures show the presence of small camerae in the internal area of centra (Fig. 6A-D). The CT scan images show a large cavity in the posterior region of the centrum, occupying almost the entire interior, surrounded by small air spaces. The anterior half of centrum, however, is composed of cancellous bone (Fig. 6E).

Figure 6. Sacral vertebrae assigned to cf. *Bonapartenykus ultimus*. A, B and C, MPCN-Pv 738.14 in anterior (A), dorsal (B) and lateral (C) views; D, MPCN-Pv 738.31 in lateral view; E, three-dimensional reconstruction of MPCN-Pv 738.31 in lateral view, and transverse (E_1), and frontal (E_2) sections. Abbreviation: **ic**, internal cavity.

The anterior and middle caudal vertebrae of cf. *Bonapartenykus ultimus* (MPCN-Pv 738.8, 10, 29, 30, 37 and 38) externally show lateral depressions and rather shallow neural fossae. Lateral surface of some caudal centra bears small and elliptical foramina, and part of natural fractures show internal air-spaces. In accordance, tomographies allow to visualize that the interior of vertebrae are invaded mainly by an asymmetrical air-spaces, however this feature is variable according to the vertebrae scanned. For instance, the first caudal centrum (MPCN-Pv 738.10) is pneumatic (with camellate tissue), except for the condyle which is solid (Fig. 7A-B). By contrast, a big camera is present in the anterior caudal centrum (MPCN-Pv 738.29), with a longitudinal septum in its dorsal area (Fig. 7C-D). In the mid-caudal vertebrae (MPCN-Pv 738.30 and MPCN-Pv 738.8) CT images show cancellous bone in the interior of the centrum, including condyle (Fig. 7E-I). Remarkably, the most complete caudal vertebra (MPCN-Pv 738.8) has a large internal air-camera in the posterior area of the neural arch, above the neural canal, which bifurcates in the anterior half. This air-structure contacts an external foramen located at the base of the neural spine (Fig. 7I) and pneumatizes almost the entire neural arch. Finally, the posterior caudal centrum (MPCN-Pv 738.11) externally has a foramen on its lateral surface. However, virtual sections show that its interior is completely solid (Fig. 7J-K).

Figure 7. Caudal vertebrae assigned to cf. *Bonapartenykus ultimus*. A MPCN-Pv 738.10 in lateral view; B, three-dimensional reconstruction of MPCN-Pv 738.10 and frontal section (B_1); C MPCN-Pv 738.29 in lateral view; D, three-dimensional reconstruction of MPCN-Pv 738.29 and frontal sections (D_1-D_2); E MPCN-Pv 738.30 in lateral view; F, three-dimensional reconstruction of MPCN-Pv 738.30 and transverse section (F_1); G, MPCN-Pv 738.8 in lateral view; H, three-dimensional reconstruction of MPCN-Pv 738.8 in lateral view, and transverse sections (H_1-H_2), and frontal sections (H_4-H_6); I, three-dimensional reconstruction of MPCN-Pv 738.8 in anterior view, and parasagittal section (I_1); J, MPCN-Pv 738.11 in lateral view; K, three-dimensional reconstruction of MPCN-Pv 738.11, and frontal section (K_1). Abbreviation: **nc**, neural canal.

Discussion

Among theropods, alvarezsaurids exhibit a distinctive skeletal pattern. Thus, members of this clade were characterized by a gracile skull with small teeth, that likely did not exceed 1 centimeter in length; large and rounded orbits; absence of contact between the postorbital and jugal bones; markedly shorter forelimbs with a robust digit; elongated hindlimbs; and hyper-elongated tails with procoelous caudal vertebrae, among other features. These morphological features have provided valuable insights that have motivated an important number of researches on several paleobiological aspects of this group [e.g., 5, 10, 39–44]. Nevertheless, among them, the pneumatic system of the postcranial skeleton has never been investigated in detail. Therefore, this study presents the first contribution focusing on the pneumatic structures of an alvarezsaurid theropod (i.e., *Bonapartenykus*), which makes use of CT scan images.

In anatomical terms, it has been established that the presence of cortical foramina may be linked to pneumatic diverticula or vasculature [17, 20]. However, the persistence of cortical foramina communicating with internal cavities (e.g., camerate tissue) in the axial skeleton is a direct correlate of the presence of pneumatic diverticula [22]. Thus, irrespective of the ontogenetic stage, the specimens studied show unambiguous evidence of postcranial pneumaticity. Here, the important value of CT images studies is highlighted here. This premise makes sense, as example, when studying specimens that present cortical foramina although their internal structure is solid, as is the case of the distal caudal vertebra (MPCN-Pv 738.8). The evidence reinforces that inferences should not be based solely on external osteological correlates. A substantial dataset of CT images from various taxa will be essential to assess the distribution of pneumatic features across alvarezaurians and theropods more broadly, as well as to further explore their paleobiological significance.

The CT images of the presacral vertebrae of *Bonapartenykus ultimus* show, in general terms, a pattern of internal air-spaces. Pneumaticity of cervical and anterior dorsal vertebrae is widespread in theropods [25]. In this aspect, presacral vertebrae of MPCN-PV 738 show camellate internal p[illegible]rn described for most other coelurosaurs, including early branching avialans such as *Rahonavis* [28, 45] and even the early diverging alvarezsaurian *Shishugounykus inexpectus* [33]. The particular case of the dorsal vertebra of the holotype specimen of *B. ultimus* (MPCA 1290) shows no signs of unambiguous pneumaticity; however, observations of this specimen are currently limited to its external anatomy only.

Furthermore, specimens studied here show tha[illegible]cral region of *Bonapartenykus* was pneumatized, as occurs in at least some abelisauroids and several tetanurans, such as spinosauroids, allosauroids, tyrannosauroids, ornithomimids, and maniraptorans, such as

alvarezsaurians [25]. Conversely, apneumatic sacral vertebrae are found in some theropods, such as the carcharodontosaurid *Tyrannotitan* [46], and the therizinosaurid *Nothronychus* [47, 48]; and, although rarely, in some birds [21].

Caudal pneumaticity has been widely recorded among theropods such as Carcharodontosauridae, Megaraptora, Ornithomimosauria, Therizinosauroidea, Oviraptorosauria and early diverging alvarezsaurians [17, 33, 34, 48]. Although several clades exhibit caudal pneumaticity, the specimens studied here show that the caudal series of *Bonapartenykus* (though incomplete and represented by disarticulated vertebrae) was pneumatized in both the anterior and middle sections. In other words, the pneumatic structures extend into the middle section of the tail. This feature is reminiscent of some Ornithomimosauria members, whose pneumaticity reached the mid-caudal vertebrae [32]. By contrast, caudal pneumaticity in theropods like carcharodontosaurids [17], therizinosaurs [49, 50], and abelisaurids are typically limited to the anterior caudals, while noasaurids exhibit apneumatic caudal vertebrae [35, 51]. Lastly, in oviraptorosaurs, caudal pneumatization would have reached the posterior caudal vertebrae [52, 53, 35]. The evidence collected so far shows that the caudal region of the alvarezsaurids showed a wide development of pneumaticity. Of particular interest is the presence of a caudal vertebra of a parvicursorine from the Hell Creek Formation with an extensive development of pneumatic structures, and based on external features, possibly greater than South American forms, including *Bonapartenykus* (JGM pers. obs.).

The study of axial elements pneumaticity within Alvarezsauria is limited to external anatomy (such as cortical foramina and natural fractures) and CT scans performed on *Shishugounykus* [33] and new specimens assigned to *Bonapartenikus*. It appears that the earlier-diverging members of Alvarezsauria, such as *Shishugounykus inexpectus*, exhibit a greater degree of pneumatic invasion compared to more derived

forms. However, the absence of cortical foramina in the Asiatic form makes the internal air spaces of the vertebrae ambiguous evidence of postcranial pneumaticity. Thus, here we highlight the first unambiguous evidence of postcranial pneumaticity in an alvarezsaurian theropod based on specimens of *Bonapartenykus*.

It is generally observed that the axial skeleton of alvarezsaurians possesses internal air spaces, in most cases accompanied by cortical foramina. In addition, *Bannykus*, *Xiyunykus* and alvarezsaurids share the presence of a foramen that develops above the neural canal in caudal vertebrae. CT images show that, at least in *Bonapartenikus*, this foramen penetrates the neural arch, a feature possibly shared by the other taxa. The data collected from alvarezsaurids does not suggest a linear evolutionary trajectory, but rather points to degree of seemingly random variability in the pneumatic features of the axial skeleton (Fig. 8). Future studies, particularly CT-based analyses of additional alvarezsaurid specimens, will be essential to fully understand the extent of pneumatic invasion among members of this clade to further comment of its macroevolution.

Figure 8. Mapped pneumatic features in the studies by Meso et al. [14]. The studied features are as follows: lateral surface of cervical centra (Ch 1; 0, simple depression; 1, foramina); internal anatomy of cervical centra (Ch 2; 0, absent; 1, present); lateral surface of dorsal centra (Ch 3; 0, simple depression; 1, foramina); internal anatomy of dorsal centra (Ch 4; 0, absent; 1, present); lateral surface of sacral centra (Ch 5; 0, simple depression; 1, foramina); internal anatomy of sacral centra (Ch 6; 0, absent; 1, present); lateral surface of caudal centra (Ch 7; 0, simple depression; 1, foramina); internal anatomy of caudal centra (Ch 8; 0, absent; 1, present); foramen developing above the neural canal in caudal vertebrae (Ch 9; 0, absent; 1, present).

The specimens referred to *Bonapartenykus* examined show a wide range of sizes and ontogenetic stages, indicating a diversity of specimens [14]. For instance, cervical neural arch (MPCN-Pv 738.28) has rough neurocentral sutures. This rough aspect of the cervical neural arch base suggests unfused nature with its vertebral centrum and is interpreted as a sign of immaturity. Additionally, the degree of pneumatic invasion within the vertebrae varies considerably between different specimens (possibly linked to ontogenetic stages) particularly in regions, such as the tail.

The described specimens have a seemingly random and asymmetrical distribution of foramina and internal pneumatic patterns (as shown by CT scans) along the axial skeleton. This lack of symmetry aligns with the idea proposed by Witmer [54] that the pneumatization process may have been largely opportunistic, potentially stemming from the emergence of pneumatic diverticula due to blood vessels, as suggested by Taylor and Wedel [24]. The random or chaotic internal pneumatization is reminiscent of the internal structure of some saurischians, as a rebbachisaurid caudal vertebra described by Windholz et al. [55]. The pneumaticity is variable because pneumatic diverticula followed blood vessels as they developed, and blood vessels are already inherently variable. The nature of pneumatization adds a second layer of variability on top of the variability imposed by the blood vessels in the first place. In other words, if blood vessels typically show some degree of variability, then It should expect that any pneumatic systems built on a pattern established by the blood vessels would be even more variable, because the process of pneumatization is opportunistic. Thus, the highly variable morphology of the internal air spaces in *Bonapartenykus* is another line of evidence that they are pneumatic rather than vascular.

The occurrence of pneumatic structures in cervical vertebrae and most-anterior dorsal vertebrae supports the presence of cervical air-sac diverticula in *Bonapartenykus*. Furtenmore, pneumatic chambers in the neural arch of posterior cervical vertebra (MPCN-Pv 738.26) connect to the neural canal, which supports the presence of paramedullary diverticula, like those of birds. Similarly, sacral and caudal pneumaticity indicate the presence of abdominal air-sac diverticula. Unfortunately, it is not possible to elucidate the presence of other air-sacs because they do not leave osteological correlates (e.g., caudal thoracic air-sac), or they pneumatize elements not preserved among the specimens here studied (e.g., sternum, sternal ribs) [25]. Finally, while reduced bone mass is advantageous for flight in modern birds, its adaptive significance in non-avian dinosaurs, including theropods of varying sizes, remains uncertain [32]. This raises the future need for further studies of this nature in the different lineages of saurischians.

Conclusions

This paper presents the first research focusing on the pneumatic features of an alvarezsaurid theropod, in this case *Bonapartenykus ultimus*. It should be noted that the axial skeleton of this species, and possibly other alvarezsaurids, was pneumatized. This premise is supported by the presence of cortical openings communicating with internal air spaces in the vertebral elements. The axial skeleton of *B. ultimus* was apparently pneumatized from the neck to the mid-tail region. Members of Alvarezsauridae show not a linear evolutionary trend, but rather show to degree of random variability in the pneumatic structures of the axial series. As concluding remarks, it emphasizes the need for studies related to the pneumatic system to examine both the external and internal anatomy of the specimens. As future perspectives, new specimens must be analyzed using new technologies (as computed tomographic images) will be necessary to fully

understand the extent of pneumatic invasion among alvarezsaurids to investigate its macroevolution trends.

Acknowledgements

We want to thank P. Chafrat from Museo Patagónico de Ciencias Naturales, General Roca, Río Negro Province, Argentina. The authors gratefully acknowledge to "Fundacion Patagonica de Ciencias Naturales" and "Sanatorio Juan XXIII", who made the materials tomographies posible. MP is supported by the School of Life Sciences of The Chinese University of Hong Kong.

References

1. Bonaparte JF. Los vertebrados fósiles de la Formación Río Colorado, de la Ciudad de Neuquén y cercanías, Cretácico Superior, Argentina. Revista del Museo Argentino de Ciencias Naturales. 1991; 4: 17–123.
2. Perle A, Chiappe LM. Barsbold R. Skeletal morphology of *Mononykus olecranus* (Theropoda: Avialae) from the late Cretaceous of Mongolia. American Museum Novitates, 1994; 3105: 1–29.
3. Novas FE. Alvarezsauridae, Cretaceous basal birds from Patagonia and Mongolia. Memoirsqueensland Museum, 1996; 39: 675–702.
4. Martinelli AG, Vera EI. *Achillesaurus manazzonei*, a new alvarezsaurid theropod (Dinosauria) from the Late Cretaceous Bajo de la Carpa Formation, Río Negro Province, Argentina. Zootaxa, 2007; 1582: 1–17.
5. Longrich NR, Currie PJ. *Albertonykus borealis*, a new alvarezsaur (Dinosauria: Theropoda) from the Early Maastrichtian of Alberta, Canada: Implications for the

systematics and ecology of the Alvarezsauridae. Cretaceous Research, 2009; 30: 239–252.

6. Agnolín FL, Powell JE, Novas FE, Kundrát M. New alvarezsaurid (Dinosauria, Theropoda) from uppermost Cretaceous of north-western Patagonia with associated eggs. Cretaceous Research, 2012; 35: 33–56.
7. Makovicky PJ, Apesteguía S, Gianechini FA. A new coelurosaurian theropod from the Lan Buitrera fossil locality of Río Negro, Argentina. Fieldiana Life and Earth Sciences, 2012; 2012: 90–98.
8. Pittman M, Xu X, Stiegler JB. The taxonomy of a new parvicursorine alvarezsauroid specimen IVPP V20341 (Dinosauria: Theropoda) from the Upper Cretaceous Wulansuhai Formation of Bayan Mandahu, Inner Mongolia, China. PeerJ, 2015; 3: e986.
9. Averianov A, Sues HD. The oldest record of Alvarezsauridae (Dinosauria: Theropoda) in the Northern Hemisphere. Plos one, 2017; 12: e0186254.
10. Fowler DW, Wilson JP, Fowler EA, Noto CR, Anduza D, Horner JR. *Trierarchuncus prairiensis* gen. et sp. nov., the last alvarezsaurid: Hell Creek Formation (uppermost Maastrichtian), Montana. Cretaceous Research, 2020; 116: 104560.
11. Qin Z, Zhao Q, Choiniere JN, Clark JM, Benton MJ, Xu X. Growth and miniaturization among alvarezsauroid dinosaurs. Current Biology, 2021; 31: 3687–3693.
12. Makovicky PJ, Apesteguia S, Gianechini FA. A new, almost complete specimen of *Alnashetri cerropoliciensis* (Dinosauria: Theropoda) impacts our understanding of alvarezsauroid evolution. Ameghiniana, 2016; 53: 6.

13. Novas FE. Anatomy of *Patagonykus puertai* (Theropoda, Avialae, Alvarezsauridae), from the Late Cretaceous of Patagonia. Journal of Vertebrate Paleontology, 1997; 17: 137–166.
14. Meso JG, Pol D, Chiappe L, Qin Z, Diaz-Martínez I, Gianechini F, Apesteguia S, Makovycky PJ, Pittman M. Body size and evolutionary rate analyses reveal complex evolutionary history of Alvarezsauria. Cladistics. In press.
15. Salgado L, Coria RA, Arcucci AB, Chiappe LM. Restos de Alvarezsauridae (Theropoda, Coelurosauria) en la Formación Alien (Campaniano-Maastrichtiano), en Salitral Ojo de Agua, Provincia de Río Negro, Argentina. Andean geology, 2009; 36: 67–80.
16. Meso JG, Choiniere J, Baiano MA, Brusatte SL, Canale JI, Salgado L, Pol D, Pittman M. New information on *Bonapartenykus* (middle Campanian-lower Mastrichtian) of Rio Negro Province, Patagonia, Argentina clarifies the Patagonykinae body plan. Plos One. In press.
17. Britt BB. Pneumatic postcranial bones in dinosaurs and other archosaurs. Doctoral thesis, University of Calgary, 1993; 4580–4580.
18. Britt BB. Postcranial pneumaticity. In: Currie PJ, Padian K, editors. The encyclopedia of dinosaurs. Oxford, UK: Academic Press, 1997; 590–593.
19. Wedel MJ. Vertebral pneumaticity, air sacs, and the physiology of sauropod dinosaurs. Paleobiology, 2003; 29: 243–255.
20. Wedel MJ. Postcranial skeletal pneumaticity in sauropods and its implications for mass estimates. In: Wilson JA, Curry-Rogers K, editors. The sauropods: evolution and paleobiology. Berkeley: University of California Press, 2005; 201–228.

21. Wedel MJ. Evidence for bird-like air sacs in saurischian dinosaurs. Journal of Experimental Zoology, 2009; 311A: 611e628.

22. O'Connor PM. Postcranial pneumaticity: an evaluation of soft-tissue influences on the postcranial skeleton and the reconstruction of pulmonary anatomy in archosaurs. Journal of Morphology, 2006; 267: 1199–1226.

23. O'Connor PM. Evolution of archosaurian body plans: skeletal adaptations of an air-sac-based breathing apparatus in birds and other archosaurs. Journal of Experimental Zoology. Part A, Ecological Genetics and Physiology, 2009; 311: 629–646.

24. Taylor M, Wedel M. Why is vertebral pneumaticity in sauropod dinosaurs so variable? Qeios, 2021; 4: 1–13.

25. O'Connor PM, Claessens LP. Basic avian pulmonary design and flowthrough ventilation in non-avian theropod dinosaurs. Nature, 2005; 253–256.

26. Yates AM, Wedel MJ, Bonnan MF. The early evolution of postcranial skeletal pneumaticity in sauropodomorph dinosaurs. Acta Palaeontologica Polonica, 2012; 57: 85–100.

27. Windholz GJ, Coria RA, Zurriaguz VL. Vertebral pneumatic structures in the Early Cretaceous sauropod dinosaur *Pilmatueia faundezi* from northwestern Patagonia, Argentina. Lethaia, 2019; 53: 369–381.

28. Gianechini FA, Zurriaguz VL. Vertebral pneumaticity of the paravian theropod *Unenlagia comahuensis*, from the Upper Cretaceous of Patagonia, Argentina. Cretaceous Research, 2021; 127: 104925.

29. Cerda IA, Salgado L, Powell JE. Extreme postcranial pneumaticity in sauropod dinosaurs from South America. Paläontologische Zeitschrift, 2012; 86: 441–449.

30. Windholz GJ, Carballido JL, Coria RA, Zurriaguz VL, Rauhut OW. How pneumatic were the presacral vertebrae of dicraeosaurid (Sauropoda: Diplodocoidea) dinosaurs? Biological Journal of the Linnean Society, 2022; 138: 103–120.
31. Benson RB, Butler RJ, Carrano M,T, O'Connor PM. Air-filled postcranial bones in theropod dinosaurs: physiological implications and the 'reptile'–bird transition. Biological Reviews, 2012; 87: 168–193.
32. Watanabe A, Leone Gold ME, Brusatte SL, Benson RB, Choiniere J, Davidson A, Norell MA. Vertebral pneumaticity in the ornithomimosaur *Archaeornithomimus* (Dinosauria: Theropoda) revealed by computed tomography imaging and reappraisal of axial pneumaticity in Ornithomimosauria. PloS one, 2015; 10(12), e0145168.
33. Qin Z, Clark J, Choiniere J, Xu X. A new alvarezsaurian theropod from the Upper Jurassic Shishugou Formation of western China. Scientific Reports, 2019; 9: 11727.
34. Aranciaga Rolando M, Garcia Marsa J, Novas F. Histology and pneumaticity of *Aoniraptor libertatem* (Dinosauria, Theropoda), an enigmatic mid-sized megaraptoran from Patagonia. Journal of Anatomy, 2020; 237: 741–756.
35. Baiano MA, Coria RA, Chiappe LM, Zurriaguz V, Coria L. Osteology of the axial skeleton of *Aucasaurus garridoi*: phylogenetic and paleobiological inferences. PeerJ, 2023; 11: e16236.
36. Wilson JA. A nomenclature for vertebral laminae in sauropods and other saurischian dinosaurs. Journal of vertebrate Paleontology, 1999; 19: 639–653.

37. Wilson JA. New vertebral laminae and patterns of serial variation in vertebral laminae of sauropod dinosaurs. Contributions from the Museum of Paleontology, University of Michigan, 2012; 32: 91–110.
38. Wilson JA, D'Emic MD, Ikejiri T, Moacdieh EM, Whitlock JA. A nomenclature for vertebral fossae in sauropods and other saurischian dinosaurs. PLoS One, 2011; 6: e17114.
39. Chiappe LM, Norell MA, Clark JM. The Cretaceous, short-armed Alvarezsauridae: *Mononykus* and its kin. Mesozoic birds: above the heads of dinosaurs, 2002; 87–120.
40. Senter P. Function in the stunted forelimbs of *Mononykus olecranus* (Theropoda), a dinosaurian anteater. Paleobiology, 2005; 31: 373–381.
41. Choiniere JN, Clark JM, Norell MA, Xu X. Cranial osteology of *Haplocheirus sollers* Choiniere et al., 2010 (Theropoda: Alvarezsauroidea). American Museum Novitates, 2014; 2014: 1–44.
42. Choiniere JN, Neenan JM, Schmitz L, Ford DP, Chapelle KE, Balanoff AM, Simpla JS, Georgi JA, Walsh SA, Norell MA, Xu X, Clark JM, Benson RB. Evolution of vision and hearing modalities in theropod dinosaurs. Science, 2021; 372: 610–613.
43. Meso JG, Qin Z, Pittman M, Canale JI, Salgado L, Díaz VD. Tail anatomy of the Alvarezsauria (Theropoda, Coelurosauria), and its functional and behavioural implications. Cretaceous Research, 2021; 124: 104830.
44. Qin Z, Liao CC, Benton MJ, Rayfield EJ. Functional space analyses reveal the function and evolution of the most bizarre theropod manual unguals. Communications Biology, 2023; 6; 1–181.

45. Forster CA, O'connor PM, Chiappe LM, Turner AH. The osteology of the Late Cretaceous paravian *Rahonavis ostromi* from Madagascar. Palaeontologia Electronica, 2020; 23:a29.

46. Novas FE, de Valais S, Vickers-Rich P, Rich T. A large Cretaceous theropod from Patagonia, Argentina, and the evolution of carcharodontosaurids. Naturwissenschaften, 2005; 92: 226–230.

47. Kirkland JI, Wolfe DG. First definitive therizinosaurid (Dinosauria; Theropoda) from North America. Journal of Vertebrate Paleontology, 2001; 21: 410–414.

48. Smith DK, Sanders RK, Wolfe DG. Vertebral pneumaticity of the North American therizinosaur *Nothronychus*. Journal of Anatomy, 2021; 238: 598–614.

49. Zanno LE, Gillette DD, Albright LB, Titus AL. A new North American therizinosaurid and the role of herbivory in 'predatory'dinosaur evolution. Proceedings of the Royal Society B: Biological Sciences, 2009; 276: 3505–3511.

50. Zanno LE. Osteology of *Falcarius utahensis* (Dinosauria: Theropoda): characterizing the anatomy of basal therizinosaurs. Zoological Journal of the Linnean Society, 2010; 158: 196–230.

51. Carrano MT, Loewen MA, Sertich JJ. New materials of *Masiakasaurus knopfleri* Sampson, Carrano, and Forster, 2001, and implications for the morphology of the Noasauridae (Theropoda: Ceratosauria). Smithsonian Contributions to Paleobiology, 2011; 95: 1–53.

52. Xu X, Tan Q, Wang J, Zhao X, Tan L. A gigantic bird-like dinosaur from the Late Cretaceous of China. Nature, 2007; 447: 844–847.

53. Balanoff AM, Norell MA. Osteology of *Khaan mckennai* (Oviraptorosauria: Theropoda). Bulletin of the American Museum of Natural History, 2012; 2012: 1–77.

54. Witmer LM. The evolution of the antorbital cavity of archosaurs: a study in soft-tissue reconstruction in the fossil record with an analysis of the function of pneumaticity. Journal of Vertebrate Paleontology, 1997; 17: 1–76.

55. Windholz GJ, Porfiri JD, Dos Santos D, Bellardini F, Wedel M.J. A well-preserved vertebra provides new insights into rebbachisaurid sauropod caudal anatomical and pneumatic features. Acta Palaeontologica Polonica, 2024; 69: 39–47.

Click here to access/download;Figure;Figure 1.tif

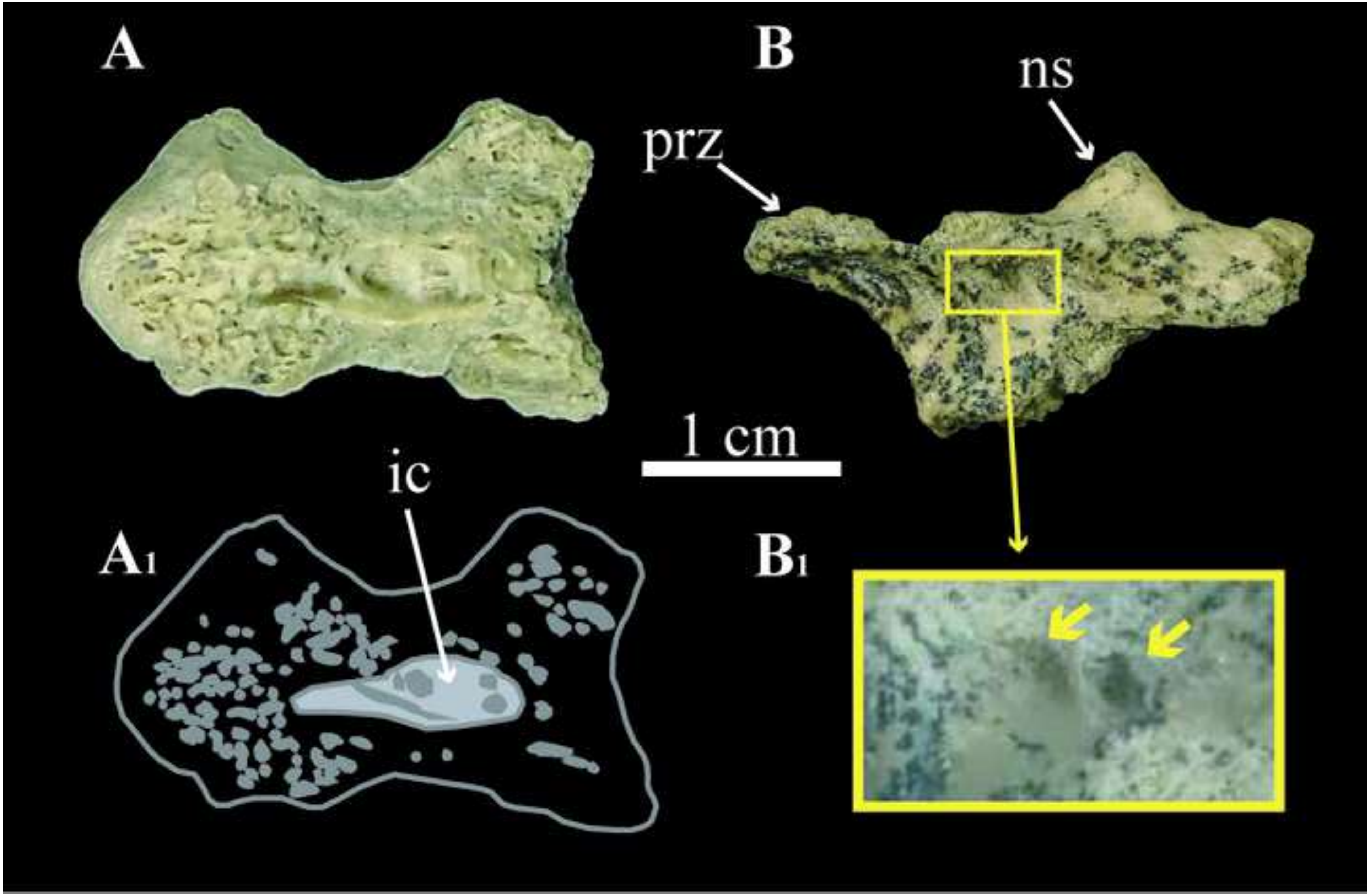

Figure 2

Click here to access/download;Figure;Figure 2.tif

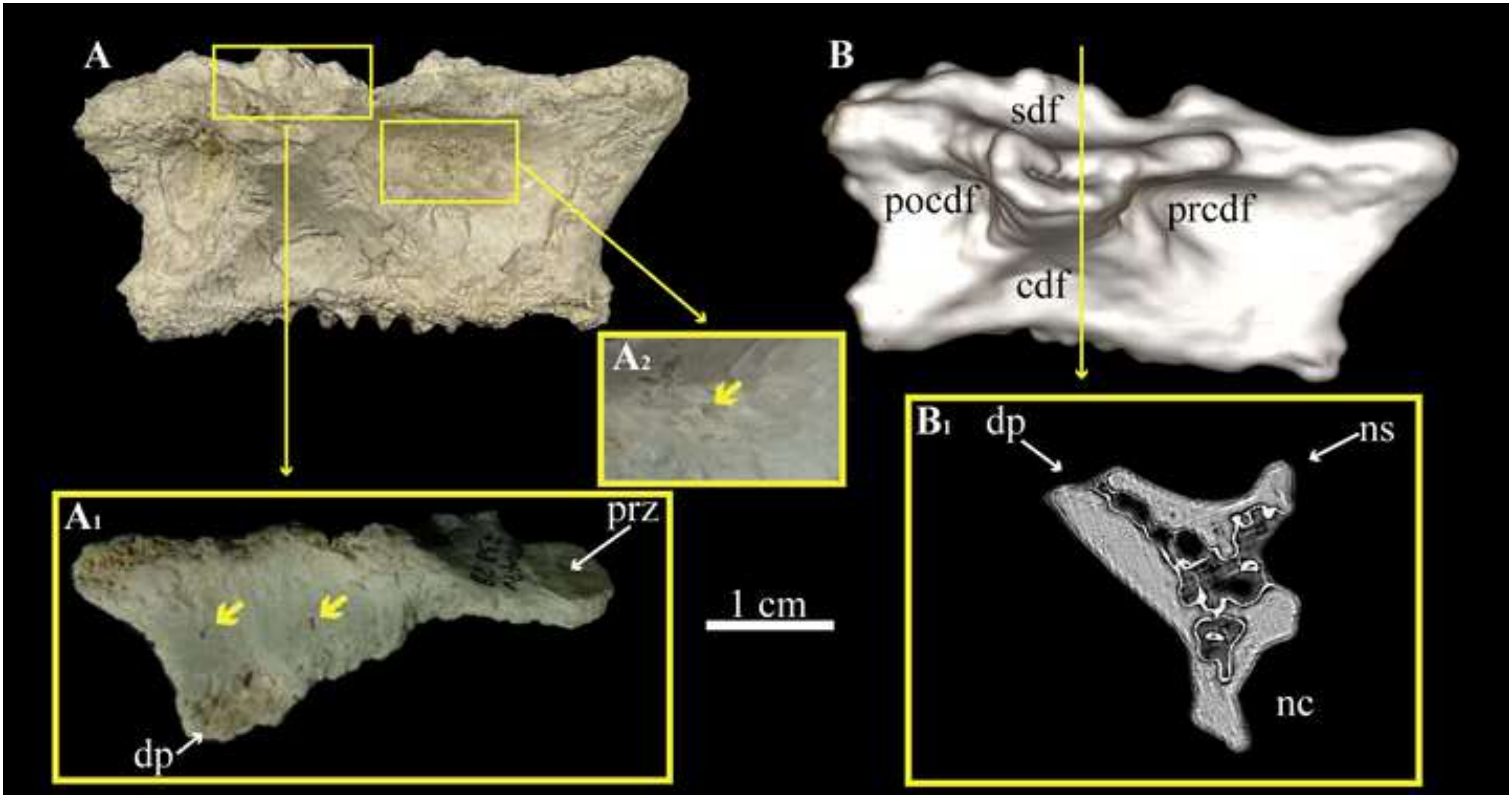

Figure 3

Click here to access/download;Figure;Figure 3.tif

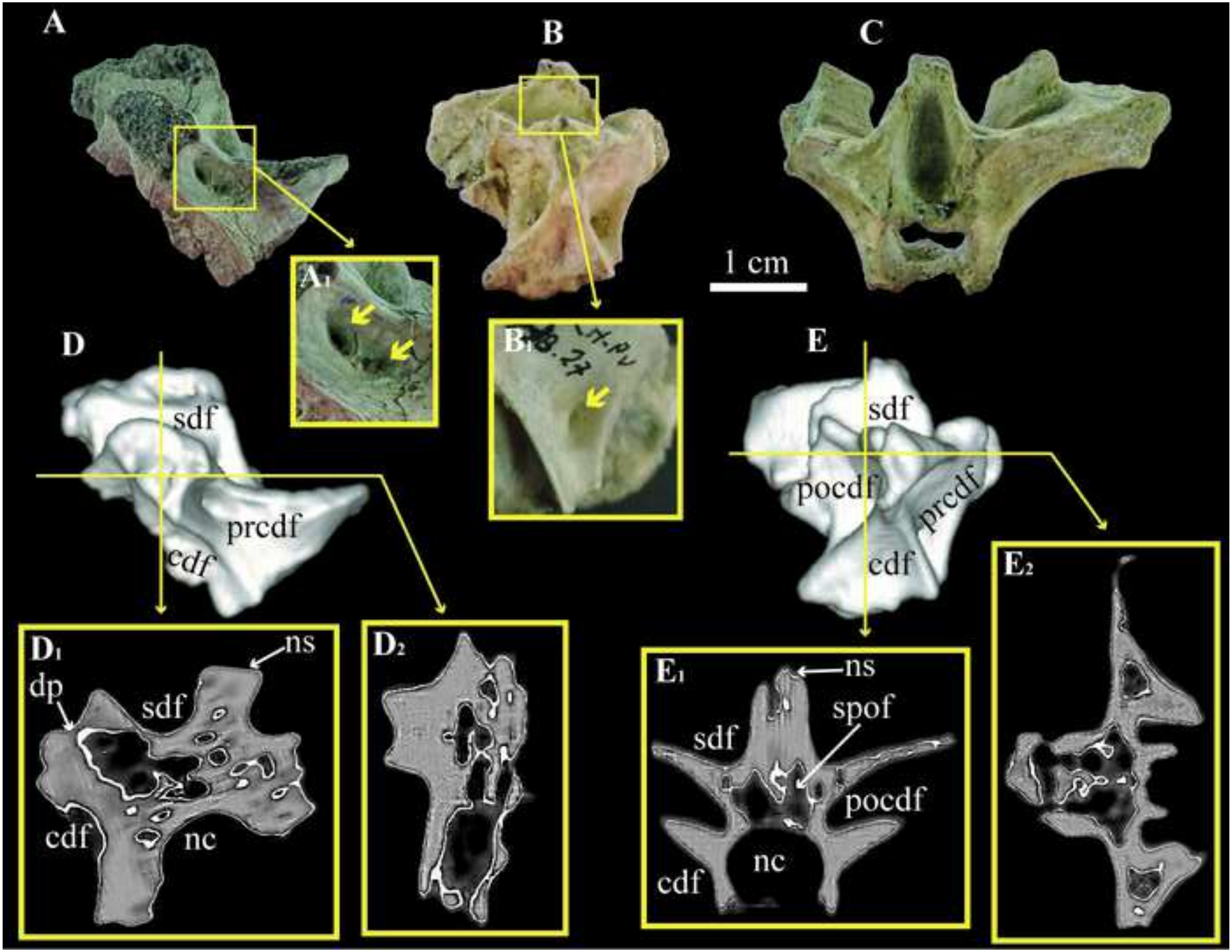

Figure 4

Click here to access/download;Figure;Figure 4.tif

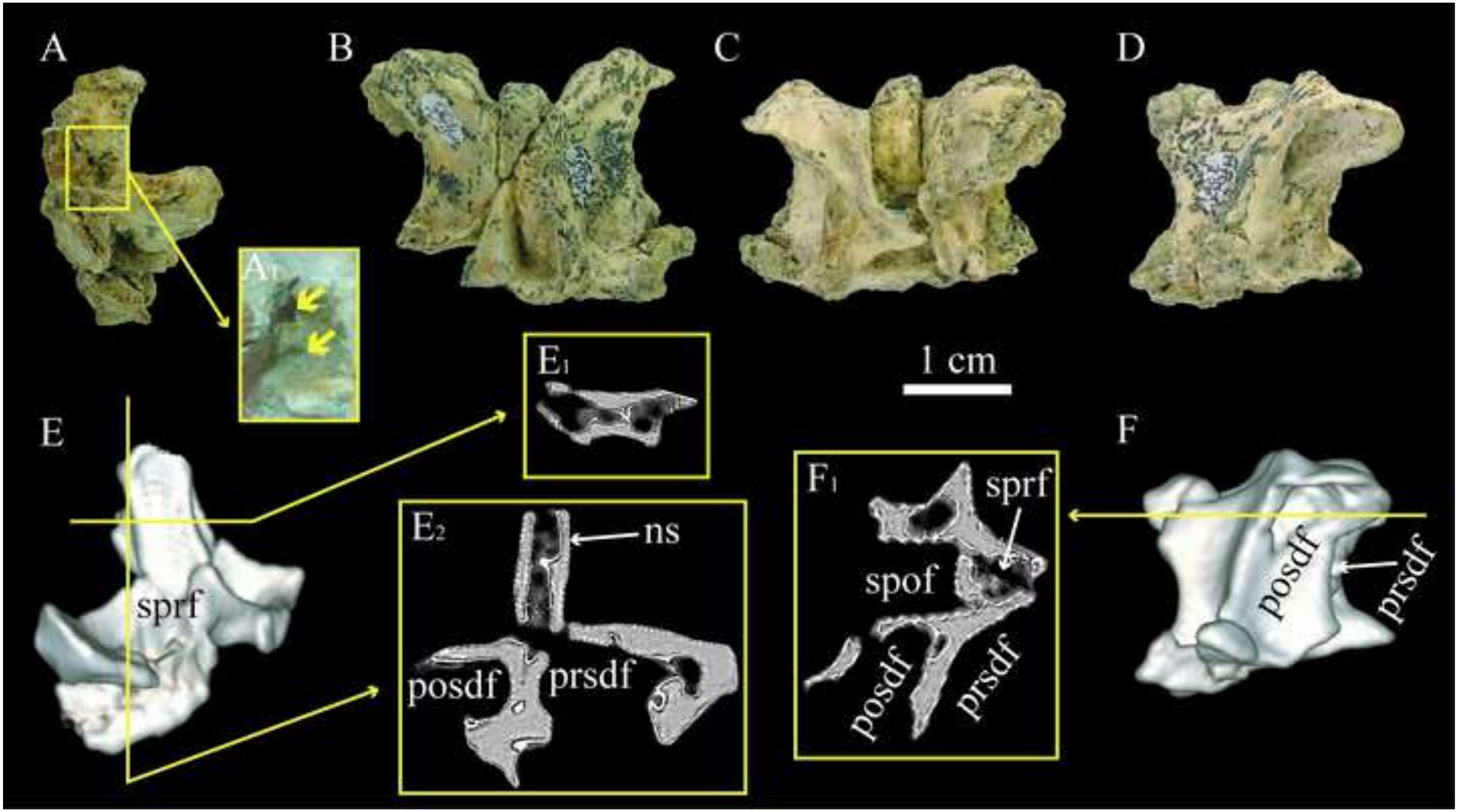

Figure 5

Click here to access/download;Figure;Figure 5.tif

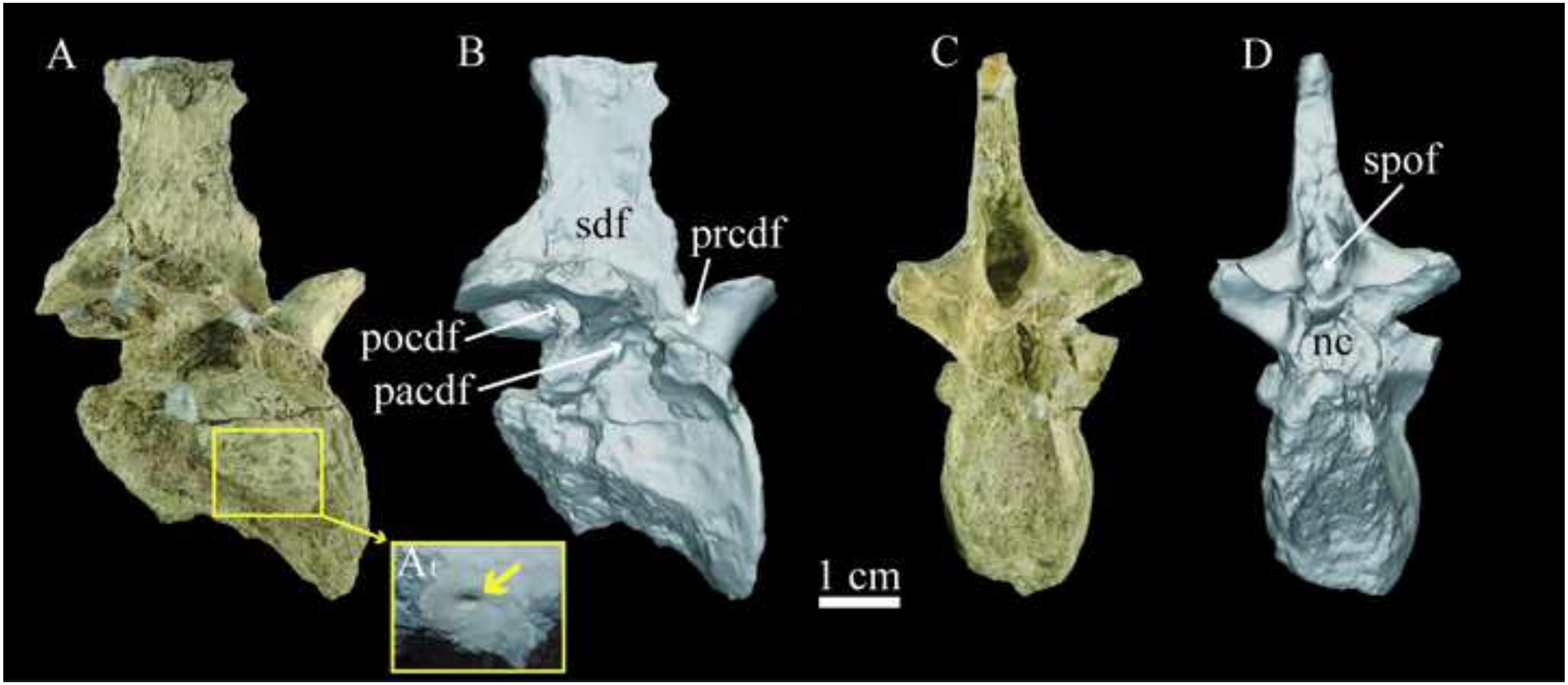

Click here to access/download;Figure;Figure 6.tif

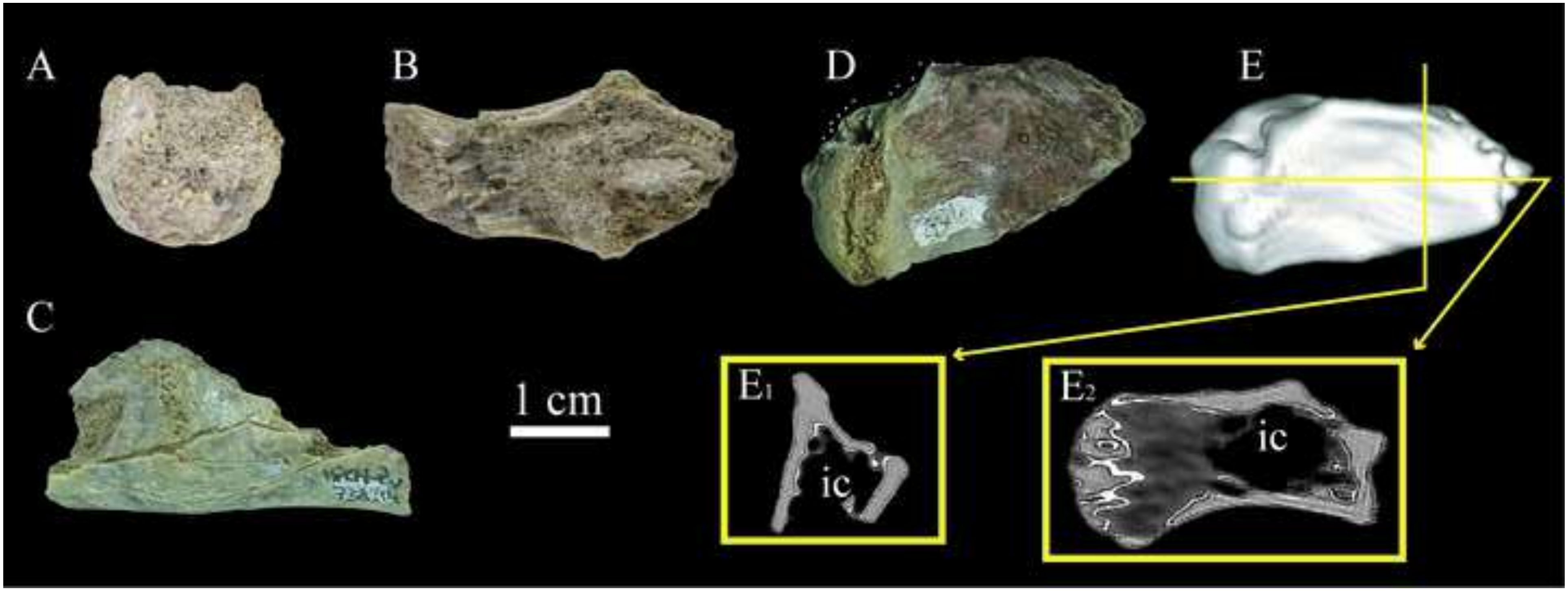

Figure 7

Click here to access/download;Figure;Figure 7.tif

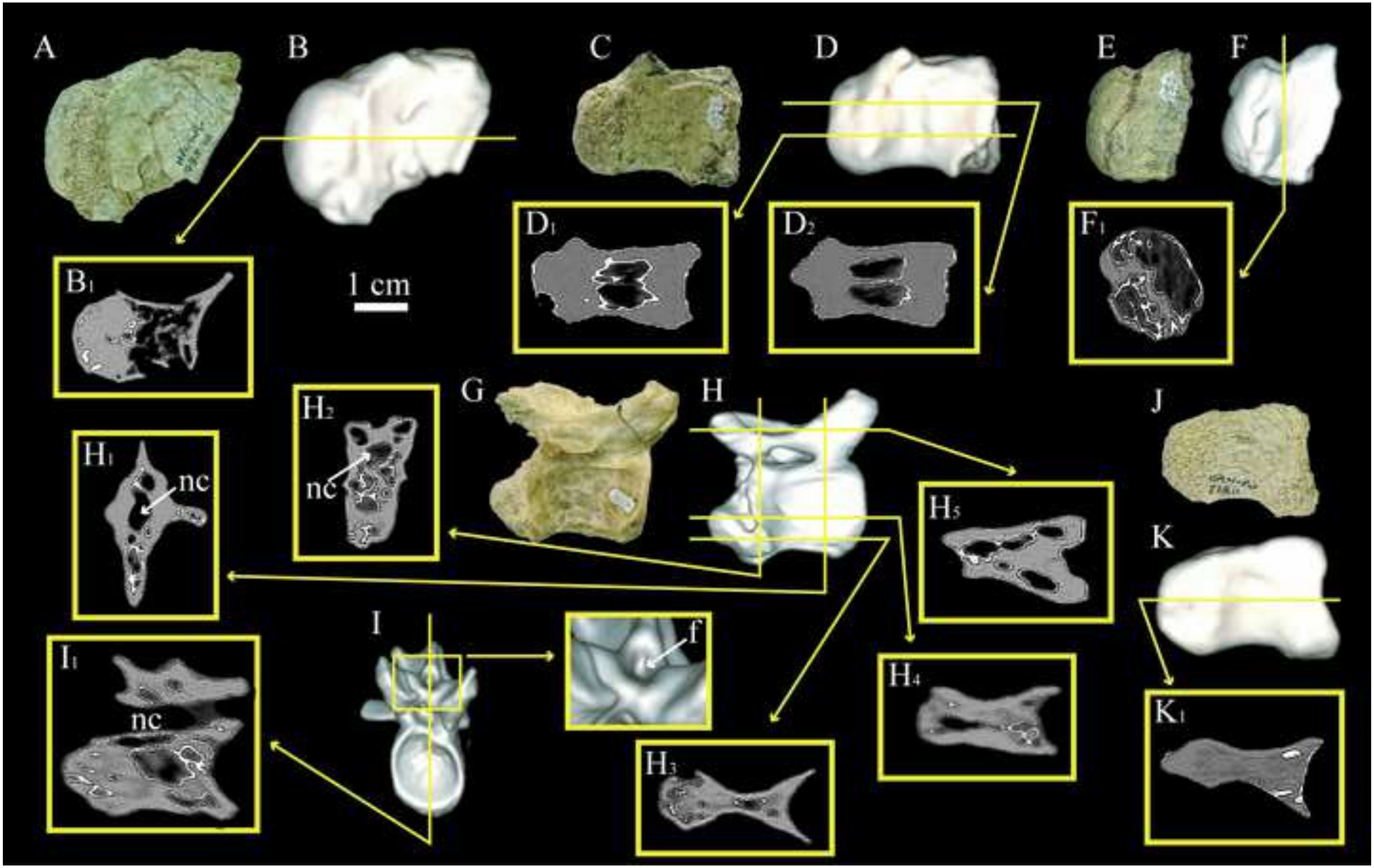

Figure 8

Click here to access/download;Figure;Figure 8.tif

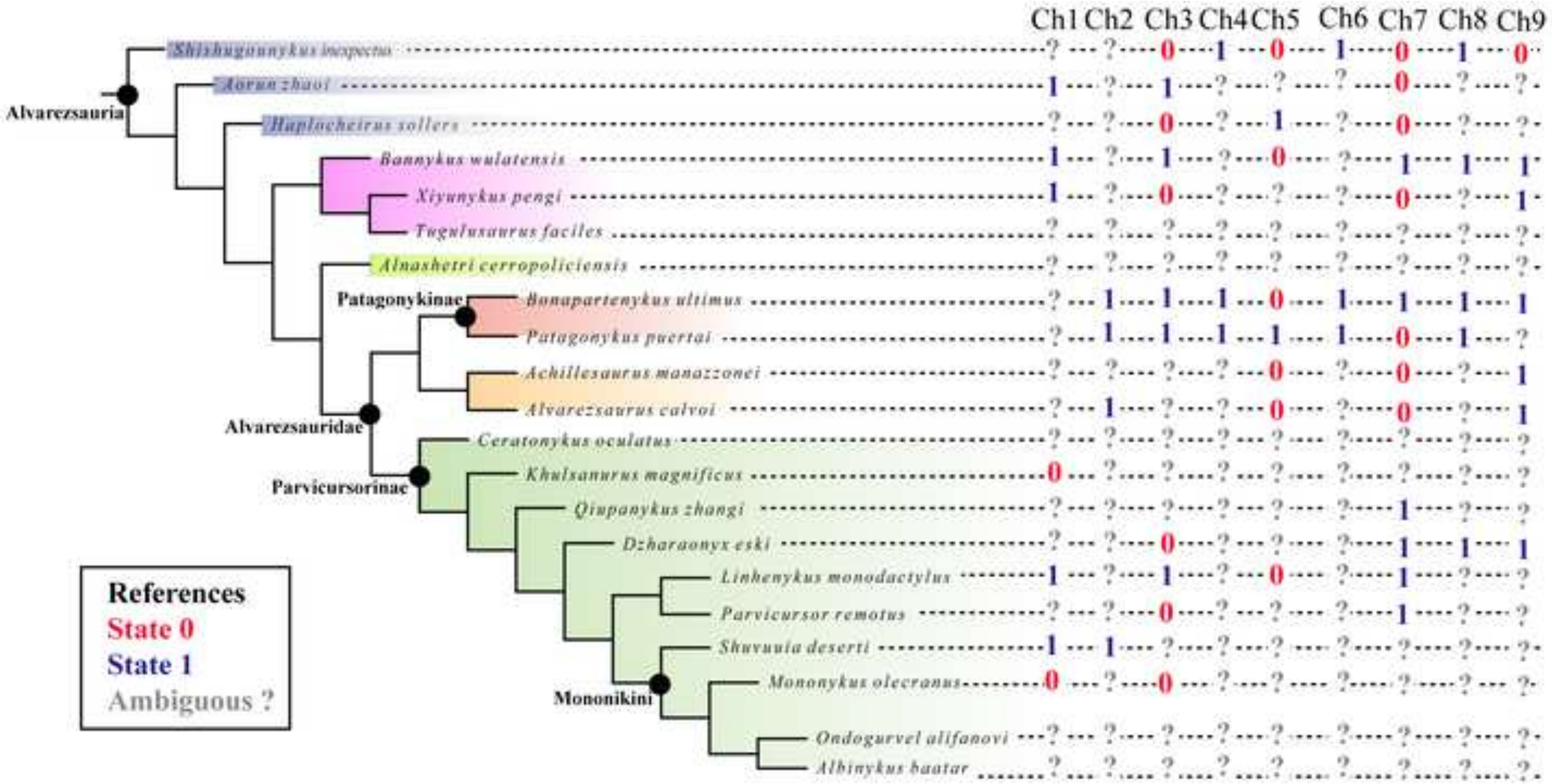

Supplement: Attachment — Submitted filename: PONE-D-24-50106.pdf [file pone.0320121.s001.pdf]
