# Supplementary material for: First unambiguous record of pneumaticity in the axial skeleton of alvarezsaurians (Theropoda: Coelurosauria)
Source: PLoS One. 2025 Apr 2;20(4):e0320121. doi: 10.1371/journal.pone.0320121 (PMC11964243; doi:10.1371/journal.pone.0320121)

PLOS ONE

First unambiguous record of pneumaticity in the axial skeleton of alvarezsaurians (Theropoda: Coelurosauria)

--Manuscript Draft--

**Manuscript Number:**	PONE-D-24-50106
**Article Type:**	Research Article
**Full Title:**	First unambiguous record of pneumaticity in the axial skeleton of alvarezsaurians (Theropoda: Coelurosauria)
**Short Title:**	First unambiguous record of axial skeleton pneumaticity in alvarezsaurians
**Corresponding Author:**	Michael Pittman<br>The Chinese University of Hong Kong<br>Shatin, Select a state... HONG KONG
**Keywords:**	Dinosauria; Theropoda; Alvareszauridae; Pneumaticity; Cretaceous; Patagonia
**Abstract:**	Bonapartenykus ultimus is an alvarezsaurid theropod from the Upper Cretaceous of Patagonia, Argentina. This species is represented by the holotype specimen and several referred specimens, which have particular bone features, such as the presence of pneumatic structures. Pneumaticity involves the invasion of the interior of the skeleton by means of air sac diverticula. Such invasion occurs from cortical openings (foramina) that communicate with internal air spaces. Despite the existence of previous studies on pneumatic structures in theropod specimens, there are no studies focusing on the family Alvarezsauridae. Thus, the first contribution focusing on the pneumatic features of an alvarezsaurid theropod is presented here. The specimens studied show that the axial skeleton of B. ultimus was invaded by pneumatic structures, reaching the middle section of the tail. Pneumaticity, among alvarezsaurids, does not suggest a linear evolutionary trajectory; but rather show to degree of random variability in the pneumatic features of the axial elements. Finally, the importance of computed tomographic images is also emphasized here, since a study on pneumaticity cannot be limited to the external anatomy of the specimens. Future studies will be essential to fully understand the extent of pneumatic invasion among alvarezsaurids to investigate its macroevolution aspects.
**Order of Authors:**	Guillermo J Windholz
	Jorge G. Meso
	Mathew J. Wedel
	Michael Pittman
**Additional Information:**	
**Question**	**Response**
**Financial Disclosure**<br><br>Enter a financial disclosure statement that describes the sources of funding for the work included in this submission. Review the submission guidelines for detailed requirements. View published research articles from *PLOS ONE* for specific examples.<br><br>This statement is required for submission and **will appear in the published article** if the submission is accepted. Please make	The author(s) received no specific funding for this work.

Powered by Editorial Manager® and ProduXion Manager® from Aries Systems Corporation

sure it is accurate.<br><br>**Funded studies**<br>Enter a statement with the following details:<br>• Initials of the authors who received each award<br>• Grant numbers awarded to each author<br>• The full name of each funder<br>• URL of each funder website<br>• Did the sponsors or funders play any role in the study design, data collection and analysis, decision to publish, or preparation of the manuscript?<br><br>Did you receive funding for this work?	
**Competing Interests**<br><br>Use the instructions below to enter a competing interest statement for this submission. On behalf of all authors, disclose any competing interests that could be perceived to bias this work—acknowledging all financial support and any other relevant financial or non-financial competing interests.<br><br>This statement is required for submission and **will appear in the published article** if the submission is accepted. Please make sure it is accurate and that any funding sources listed in your Funding Information later in the submission form are also declared in your Financial Disclosure statement.<br><br>View published research articles from *PLOS ONE* for specific examples.	The authors have declared that no competing interests exist.

Powered by Editorial Manager® and ProduXion Manager® from Aries Systems Corporation

**NO authors have competing interests**<br><br>Enter: *The authors have declared that no competing interests exist.*<br><br>**Authors with competing interests**<br><br>Enter competing interest details beginning with this statement:<br><br>*I have read the journal's policy and the authors of this manuscript have the following competing interests: [insert competing interests here]*<br><br>* typeset	
**Ethics Statement**<br><br>Enter an ethics statement for this submission. This statement is required if the study involved:<br><br>• Human participants<br>• Human specimens or tissue<br>• Vertebrate animals or cephalopods<br>• Vertebrate embryos or tissues<br>• Field research<br><br>Write "N/A" if the submission does not require an ethics statement.<br><br>General guidance is provided below. Consult the submission guidelines for detailed instructions. **Make sure that all information entered here is included in the Methods section of the manuscript.**	N/A. Specimens studied were previously accessioned specimens in institutional repositories.

**Format for specific study types**<br><br>**Human Subject Research (involving human participants and/or tissue)**<br>• Give the name of the institutional review board or ethics committee that approved the study<br>• Include the approval number and/or a statement indicating approval of this research<br>• Indicate the form of consent obtained (written/oral) or the reason that consent was not obtained (e.g. the data were analyzed anonymously)<br><br>**Animal Research (involving vertebrate animals, embryos or tissues)**<br>• Provide the name of the Institutional Animal Care and Use Committee (IACUC) or other relevant ethics board that reviewed the study protocol, and indicate whether they approved this research or granted a formal waiver of ethical approval<br>• Include an approval number if one was obtained<br>• If the study involved *non-human primates*, add *additional details* about animal welfare and steps taken to ameliorate suffering<br>• If anesthesia, euthanasia, or any kind of animal sacrifice is part of the study, include briefly which substances and/or methods were applied<br><br>**Field Research**<br><br>Include the following details if this study involves the collection of plant, animal, or other materials from a natural setting:<br>• Field permit number<br>• Name of the institution or relevant body that granted permission	
**Data Availability**<br><br>Authors are required to make all data underlying the findings described fully available, without restriction, and from the time of publication. PLOS allows rare exceptions to address legal and ethical concerns. See the PLOS Data Policy and FAQ for detailed information.	Yes - all data are fully available without restriction

A Data Availability Statement describing where the data can be found is required at submission. Your answers to this question constitute the Data Availability Statement and **will be published in the article**, if accepted.<br><br>**Important:** Stating 'data available on request from the author' is not sufficient. If your data are only available upon request, select 'No' for the first question and explain your exceptional situation in the text box.<br><br>Do the authors confirm that all data underlying the findings described in their manuscript are fully available without restriction?	
**Describe where the data may be found in full sentences. If you are copying our sample text, replace any instances of XXX with the appropriate details.**<br><br>• If the data are **held or will be held in a public repository**, include URLs, accession numbers or DOIs. If this information will only be available after acceptance, indicate this by ticking the box below. For example: *All XXX files are available from the XXX database (accession number(s) XXX, XXX.).*<br>• If the data are all contained **within the manuscript and/or Supporting Information files**, enter the following: *All relevant data are within the manuscript and its Supporting Information files.*<br>• If neither of these applies but you are able to provide **details of access elsewhere**, with or without limitations, please do so. For example:<br><br>*Data cannot be shared publicly because of [XXX]. Data are available from the XXX Institutional Data Access / Ethics Committee (contact via XXX) for researchers who meet the criteria for access to confidential data.*<br><br>*The data underlying the results presented in the study are available from (include the name of the third party*	All relevant data are within the manuscript and its Supporting Information files.

and contact information or URL).<br>• This text is appropriate if the data are owned by a third party and authors do not have permission to share the data.<br><br>* typeset	
Additional data availability information:	

Powered by Editorial Manager® and ProduXion Manager® from Aries Systems Corporation

Manuscript

Click here to access/download;Manuscript;Windholz et al. main text_20241103.docx

First unambiguous record of pneumaticity in the axial skeleton of alvarezsaurians (Theropoda: Coelurosauria)

G.J. Windholz*,[1], J.G. Meso[1], M.J. Wedel[2], M. Pittman*,[3]

[1]CONICET- Instituto de Investigación en Paleobiología y Geología, Sede Alto Valle-Valle Medio Universidad Nacional de Río Negro, Av. Roca 1242, Gral. Roca, Río Negro, Argentina. gwindholz@unrn.edu.ar jgmeso@unrn.edu.ar

[2]College of Osteopathic Medicine of the Pacific and College of Podiatric Medicine, Western University of Health Sciences, Pomona, California, 91711, USA. mathew.wedel@gmail.com

[3]School of Life Sciences, The Chinese University of Hong Kong, Hong Kong SAR, China. mpittman@cuhk.edu.hk

*corresponding authors

Abstract

Bonapartenykus ultimus is an alvarezsaurid theropod from the Upper Cretaceous of Patagonia, Argentina. This species is represented by the holotype and several referred specimens, which have particular bone features, such as the presence of pneumatic structures. Pneumaticity involves the invasion of the interior of bones by means of air sac diverticula. Such invasion occurs from cortical openings (foramina) that open into internal air spaces. Despite the existence of previous studies on pneumatic structures in theropod specimens, there are no studies focusing on the family Alvarezsauridae. Thus, the first contribution focusing on the pneumatic features of an alvarezsaurid theropod is presented here. The specimens studied show that the axial skeleton of *B. ultimus* was invaded by pneumatic structures, extending to the middle section of the tail. Pneumaticity, among alvarezsaurids, does not suggest a linear evolutionary trajectory; but rather show to degree of random variability in the pneumatic features of the axial elements. Finally, the importance of computed tomographic images is also emphasized heresince a study on pneumaticity cannot be limited to the external anatomy of the specimens. Future studies will be essential to fully understand the extent of pneumatic invasion among alvarezsaurids and investigate its macroevolution aspects.

Key words

Dinosauria, Theropoda, Alvareszauridae, Pneumaticity, Cretaceous, Patagonia

Introduction

Alvarezsauridae is a group of early diverging maniraptoran theropod dinosaurs named by Bonaparte [1]. The fossil records of this dinosaur family come from the Upper Cretaceous of South America, North America, Europe, and Asia [2–11]. Notably, the

diversity of this clade in South America is limited to Argentina, where five species have been described to date: *Alnashetri cerropoliciensis* from the Candeleros Formation, Cenomanian [7, 12]; *Patagonykus puertai* from the Portezuelo Formation, Coniacian [3, 13]; *Alvarezsaurus calvoi* and *Achillesaurus manazzonei* from the Bajo de la Carpa Formation, Coniacian-Santonian [1, 4]; and *Bonapartenykus ultimus* from the Allen Formation, middle Campanian–lower Maastrichtian [6].

Among the Argentinian forms an endemic subclade Patagonykinae, currently including *Patagonykus puertai* and *Bonapartenykus ultimus, h*as been identified [6]. This subfamily showed a tendency toward larger body sizes, relative to other alvarezsaurians, with body lengths estimated to range from ~3 to ~3.3 meters [14]. *Bonapartenykus ultimus* comes from the "Salitral Ojo de Agua" locality, Rio Negro Province (Patagonia Argentina) and it is the only known species of the genus. The species is represented by holotype published by Agnolín et al. [6], along with several additionalspecimens referred by Salgado et al. [15] and Meso et al. [16]. These specimens exhibit a particular bony anatomy, including structures that would indicate the presence of postcranial pneumaticity.

Pneumaticity is a widely distributed feature among archosaurs, including pterosaurs and saurischian dinosaurs [17–24]. However, birds are the only living archosaurs with postcranial pneumaticity in their skeleton because crocodylians lack it [23]. The pneumatic system involves the invasion of bone by air sac diverticula, which may manifest through observable external (such as fossae, foramina, and laminae) and internal skeletal features (e.g., procamerate, camerate, and camellate). Numerous studies have explored the pneumatic system of dinosaurs [e.g., 20, 21, 25–28]. Sauropods have been the main focus of attention [e.g., 19–21, 29, 30], perhaps due to the phenomenon of gigantism, but there have also been

studies investigating pneumaticity in non-avian theropods [e.i. 22, 23, 28, 31, 32, 34, 35]. Nevertheless, to date, no studies have focused on the pneumaticity of the family Alvarezsauridae. This study aims to fill that gap by characterizing the pneumatic structures in the axial skeleton of *Bonapartenykus* based on first-hand study of external anatomy as well as both external and internal anatomy from computed tomography scans. We also compared the observed pneumatic pattern with other theropods, focusing on alvarezsaurians.

Materials and methods

The materials studied here come from the middle Upper Cretaceous (Campanian-lower Maastrichtian) Allen Formation of Río Negro Province, Argentina. The holotype of *Bonapartenykus* (MPCA 1290) is housed in the MPCA "Museo Provincial Carlos Ameghino", Cipolletti City; while referred specimens (MPCN-PV 738) are housed in the MPCN “Museo Patagonico de Ciencias Naturales”, located in General Roca City; both in Río Negro Province, Argentina.

The computed tomographic images (CTs) were obtained from eleven vertebrae: a mid-cervical vertebra (MPCN-Pv 738.28), two posterior cervical vertebrae (MPCN-Pv 738.26 and 27), two cervico-dorsal vertebrae (MPCN-Pv 738.15 and 16), a sacral vertebra (MPCN-Pv 738.14), the first caudal vertebra (MPCN-Pv 738.10), an anterior caudal vertebrae (MPCN-Pv 738.29), two mid-caudal (MPCN-Pv 738.8 and 30) and a posterior caudal vertebra (MPCN-Pv 738.11). The scans were performed at “Sanatorio Juan XXIII” hospital in General Roca, Río Negro Province, Argentina. The remaining elements were examined first-hand for external anatomical features and to a limited extent some internal anatomy accessible through natural fractures in the fossils.

The descriptions use the nomenclature vertebral laminae and fossae proposed by Wilson [36, 37] and Wilson et al. [38] as well as the terminology for pneumatic structures of Britt [17], Wedel [20] and O'Connor [22].

Results

The examination was based on two anterior cervical vertebrae assigned to cf. *Bonapartenykus ultimus* (MPCN-PV 738), a vertebral centrum (MPCN-Pv 738.32) and an incomplete neural arch (MPCN-Pv 738.47). The external surface of the anterior cervical (MPCN-Pv 738.32) lacks foramina. However, it presents a deep depression in its ventral region. This centrum is broken in its dorsal region, thus exposing a wide internal cavity surrounded by a network of interconnected trabeculae (Fig. 1A). Likewise, the neural arch (MPCN-Pv 738.47) bears poor developed laminae and shallow neural fossae (Fig. 1B). This vertebra has a pair of foramina on the inner surface of centrodiapophyseal fossa (**cdf**).

Figure 1. Anterior cervical vertebrae assigned to cf. *Bonapartenykus ultimus*. A, MPCN-Pv 738.32 in dorsal view; A_1, interpretive drawing showing internal air spaces; B, MPCN-Pv 738.47 in lateral view; B_1, detail of foramina. Abbreviations: **ic**, internal cavity; **ns**, neural spine; **prz**, prezygapophysis.

The mid-cervical region of cf. *Bonapartenykus ultimus* is represented by the vertebra (MPCN-Pv 738.28). Its neural arch has a rough appearance on its ventral surface, indicating an open neurocentral suture. This vertebra presents a pattern of poorly developed neural laminae,

accompanied by wide lateral fossae (i.e., **cdf**, **prcdf**, **pocdf** and **sdf**) (Fig. 2A). Additionally, natural fractures in the transverse processes and base of the neural spine reveal a three-dimensional network of trabeculae, with small interconnected air spaces. Particularly, the inner surfaces of the entrodiapophyseal fossa (**prcdf**) and spinodiapophyseal fossa (**sdf**) of MPCN-Pv 738.28 bear foramina (Fig. 2A). Although the mid-cervical fossae are shallow (Fig. 2B), CT-scan images show a network of internally interconnected air spaces (Fig. 2B).

Figure 2. Mid-cervical vertebrae MPCN-Pv 738.28 assigned to cf. *Bonapartenykus ultimus*. A, MPCN-Pv 738.28 in lateral view; A_1, close ups showing foramina on **sdf** and A_2, on **prcdf**; B, three-dimensional reconstruction of MPCN-Pv 738.28 in lateral view; B_1, transverse section of MPCN-Pv 738.28 at mid-length, note the internal air spaces. Abbreviations: **cdf,** centrodiapophyseal fossa; **nc**, neural canal; **ns**, neural spine; **dp**, diapophysis; **prcdf**, prezygapophyseal centrodiapophyseal fossa; **prz**, prezygapophysis; **pocdf**, postzygapophyseal centrodiapophyseal fossa; **sdf,** spinodiapophyseal fossa.

The posterior cervical vertebrae assigned to cf. *Bonapartenykus ultimus* (MPCN-Pv 738.26 and 27) preserve only the neural arches. The pattern of neural laminae and fossae is considerably more complex compared to mid-cervical vertebrae (Fig. 3A and B). The lateral fossae (i.e., **cdf**, **prcdf**, **pocdf** and **sdf**) are deep, almost reaching the midline of the element. The posterior cervical vertebra (MPCN-Pv 738.27) has a deep spinopostzygapophyseal fossa (**spof**) with an elliptical outline (Fig. 3C). This fossa extends almost to the roof of the neural canal, invading almost half of the neural arch. The **spof** is not visible on the vertebra (MPCN-Pv 738.26) since the posterior surface of the

neural spine is completely eroded. The inner surfaces of the prezygapophyseal centrodiapophyseal fossae (**prcdf**) on the posterior cervical vertebrae each have a pair of foramina in MPCN-Pv 738.26 and a simple foramen in MPCN-Pv 738.27. Additionally, both posterior cervical vertebrae have a poorly developed lamina on the inner surface of the spinodiapophyseal fossae (**sdf**) and a foramen on each side of that lamina; the only exception is the right **sdf** of MPCN-Pv 738.26, which is simple and contains only one foramen. Interestingly, CT-images also show the interior of the neural arch with interconnected air spaces, including some small chambers in the neural spine (Fig. 3D and E).

Figure 3. Posterior cervical vertebrae assigned to cf. *Bonapartenykus ultimus*. A, MPCN-Pv 738.26 in lateral view, and detail of foramina (A_1); B and C, MPCN-Pv 738.27 in lateral (B), detail of foramina (B_1), and posterior views (C); D, three-dimensional reconstruction of MPCN-Pv 738.26 in lateral view, transverse (D_1), and frontal (D_2) sections; E, three-dimensional reconstruction of MPCN-Pv 738.27 in lateral view, transverse (E_1), and frontal (E_2) sections. Abbreviations: **cdf,** centrodiapophyseal fossa; **dp**, diapophysis; **nc**, neural canal; **ns**, neural spine; **prcdf**, prezygapophyseal centrodiapophyseal fossa; **pocdf**, postzygapophyseal centrodiapophyseal fossa; **sdf** spinodiapophyseal fossa; **spof**, spinopostzygapophyseal fossa.

The cervico-dorsal transition of cf. *Bonapartenykus ultimus* is represented by two incomplete vertebrae, MPCN-Pv 738.15 and 16, which both lack the centrum, and an isolated fragment of a postzygapophysis MPCN-Pv 738.48 and a neural spine MPCN-Pv 738.49. Their pneumatic fossae (**prsdf**, **posdf**, **sprf** and **spof**) are wide and deep (Fig. 4A-D),

especially the postzygapophyseal spinodiapophyseal fossa (**posdf**) that invades almost to the axial plane of the neural arch (Fig. 4E and F). The cervicodorsal transition is characterized by the presence of deep spinoprezygapophyseal (**sprf**) and spinopostzygapophyseal (**spof**) fossae with elliptical outlines. This anatomical region is also represented by a fragment of isolated postzygapophysis (MPCN-Pv 738.48) and neural spine (MPCN-Pv 738.49), whose fractures show large internal cameras. The specimen (MPCN-Pv 738.15) has two foramina, separated by a thin bony partition on the interior surface of the prezygapophyseal spinodiapophyseal fossa (**prsdf**). CT-mages show that the interior of the neural spine of MPCN-Pv 738.15 and 16 has abundant interconnected air spaces (Fig. 4E and F).

Figure 4. Cervico-dorsal vertebrae assigned to cf. *Bonapartenykus ultimus*. A, MPCN-Pv 738.15 in lateral view, and detail of two foramina (A_1); B, C, and D, MPCN-Pv 738.16 in anterior (B), posterior (C), and lateral views (C); E, three-dimensional reconstruction of MPCN-Pv 738.15 in anterior view, frontal (E_1), and parasagittal (E_2) sections; F, three-dimensional reconstruction of MPCN-Pv 738.16 in lateral view and frontal section (F_1). Abbreviations: **ns**, neural spine; **posdf,** postzygapophyseal spinodiapophyseal fossa; **prsdf**, prezygapophyseal spinodiapophyseal fossa; **spof**, spinopostzygapophyseal fossa; **sprf**, spinoprezygapophyseal fossa.

The mid-dorsal vertebra (MPCA 1290, holotype) was almost completely preserved and has been described in detail by Agnolín et al. [6]. However, it is interesting to note that some features can be linked to the presence/ absence of pneumaticity of the only vertebra preserved in

the holotype of *Bonapartenykus ultimus*. For example, the centrum lacks pleurocoels, although it bears a foramen on its lateral surface. Natural fractures in the centrum do not appear to show signs of internal pneumaticity, such as camellae or camerae although the preservation in this region is poor. The neural arch presents well-developed lamellae that delimit wide and deep **pacdf**, **pocdf** and **sdf**; and a much reduced **prcdf** (Fig. 5A-B). The neural arch has a deep **spof** (Fig. 5C).

Figure 5. Mid-dorsal vertebrae of holotype specimen of *Bonapartenykus ultimus* (MPCA 1290). A, vertebra in lateral view and detail of foramina (A_1); B, three-dimensional reconstruction in lateral view; C, vertebral element in posterior view; D three-dimensional reconstruction in posterior view. Abbreviations: **nc**, neural canal; **pacdf**, parapophyseal centrodiapophyseal fossa; **pocdf**, postzygapophyseal centrodiapophyseal fossa; **prcdf**, prezygapophyseal centrodiapophyseal fossa; **sdf** spinodiapophyseal fossa; **spof**, spinopostzygapophyseal fossa.

The sacral region of cf. *Bonapartenykus ultimus* is represented by two anterior sacral vertebrae (MPCN-Pv 738.14) and the last sacral vertebra (MPCN-Pv 738.31). The poor state of preservation of those elements makes it difficult to observe certain features; however, natural fractures show the presence of small camerae in the internal area of centra (Fig. 6A-D). The CT-scan images show a large cavity in the posterior region of the centrum, occupying almost the entire interior, surrounded by small air spaces. The anterior half of the centrum, however, is composed of cancellous bone (Fig. 6E).

Figure 6. Sacral vertebrae assigned to cf. *Bonapartenykus ultimus*. A, B and C, MPCN-Pv 738.14 in anterior (A), dorsal (B) and lateral (C) views; D, MPCN-Pv 738.31 in lateral view; E, three-dimensional reconstruction of MPCN-Pv 738.31 in lateral view, and transverse (E_1), and frontal (E_2) sections. Abbreviation: **ic**, internal cavity.

The anterior and middle caudal vertebrae of cf. *Bonapartenykus ultimus* (MPCN-Pv 738.8, 10, 29, 30, 37 and 38) show lateral depressions and rather shallow neural fossae. The lateral surface of some caudal centra bears small and elliptical foramina, and some natural fractures show internal air-spaces. CT-scan images show that this feature is variable among the vertebrae scanned. For instance, the first caudal centrum (MPCN-Pv 738.10) is pneumatic (with camellate tissue) except for its condyle which is solid (Fig. 7A-B). By contrast, a large chamber is present in the anterior caudal centrum (MPCN-Pv 738.29), with a longitudinal septum dorsally (Fig. 7C-D). In the mid-caudal vertebrae (MPCN-Pv 738.30 and MPCN-Pv 738.8) CT-images show cancellous bone in the interior of the centrum, including the condyle (Fig. 7E-I). Remarkably, the most complete caudal vertebra (MPCN-Pv 738.8) has a large internal air chamber in the posterior area of the neural arch, above to neural canal, which bifurcates in the anterior half. This chamber connects to an external foramen located at the base of the neural spine (Fig. 7I) and invades almost the entire neural arch. Finally, the posterior caudal centrum (MPCN-Pv 738.11) has a foramen on its lateral surface. However, virtual sections show that its interior is completely solid (Fig. 7J-K).

Figure 7. Caudal vertebrae assigned to cf. *Bonapartenykus ultimus*. A MPCN-Pv 738.10 in lateral view; B, three-dimensional reconstruction of MPCN-Pv 738.10 and frontal section (B_1); C MPCN-Pv 738.29 in lateral view; D, three-dimensional reconstruction of MPCN-Pv 738.29 and frontal sections (D_1-D_2); E MPCN-Pv 738.30 in lateral view; F, three-dimensional reconstruction of MPCN-Pv 738.30 and transverse section (F_1); G, MPCN-Pv 738.8 in lateral view; H, three-dimensional reconstruction of MPCN-Pv 738.8 in lateral view, and transverse sections (H_1-H_2), and frontal sections (H_4-H_6); I, three-dimensional reconstruction of MPCN-Pv 738.8 in anterior view, and parasagittal section (I_1); J, MPCN-Pv 738.11 in lateral view; K, three-dimensional reconstruction of MPCN-Pv 738.11, and frontal section (K_1). Abbreviation: **nc**, neural canal.

Discussion

Among theropods, alvarezsaurids exhibit a distinctive skeletal structure. Thus, members of this clade are characterized by a gracile skull with small teeth, which likely did not exceed 1 cm in apicobasal height; large and rounded orbits; absence of contact between the postorbital and the jugal; remarkably short forelimbs with a single robust digit; elongated hindlimbs; and greatly elongated tails with procoelous caudal vertebrae, among other features. These morphological features have provided valuable insights into several paleobiological aspects of this group [e.g., 5, 10, 39–44]. However, the pneumatic system of the postcranial skeleton has never been investigated in detail. This study presents the first analysis focusing on the pneumatic structures of an alvarezsaurid theropod (i.e., *Bonapartenykus*), which makes use of CT-scan images.

In anatomical terms, it has been established that cortical foramina may be linked to pneumatic diverticula or vasculature [17, 20]. The persistence of cortical foramina communicating with internal cavities (e.g., camerate tissue) in the axial skeleton is a direct correlate of the presence of pneumatic diverticula [22]. Thus, irrespective of the ontogenetic stage, the specimens studied show unambiguous evidence of postcranial pneumaticity. Here, the value of CT-images studies is highlighted here. This approach is helpful when studying specimens that present cortical foramina although their internal structure is solid, as is the case for the distal caudal vertebra (MPCN-Pv 738.8). The evidence reinforces that inferences should not be based solely on external osteological correlates. A substantial dataset of CT-images from various taxa will be essential to assess the distribution of pneumatic features among alvarezaurians and theropods more broadly and to explore their paleobiological significance.

The CT-images of the presacral vertebrae of *Bonapartenykus ultimus* show, in general terms, a pattern of internal pneumatic spaces. Pneumaticity of cervical and anterior dorsal vertebrae is widespread in theropods [25]. Presacral vertebrae of MPCN-PV 738 show camellate internal pattern described for most other coelurosaurs, including early-branching avialans such as *Rahonavis* [28, 45] and even the early-diverging alvarezsaurian *Shishugounykus inexpectus* [33]. The dorsal vertebra of the holotype of *B. ultimus* (MPCA 1290) shows no signs of unambiguous pneumaticity; however, observations of this specimen are currently limited to its external anatomy.

Furthermore, specimens studied here show that sacral region of *Bonapartenykus* was pneumatized, as in at least some abelisauroids and several tetanurans such as spinosauroids, allosauroids, tyrannosauroids, ornithomimids, and maniraptorans such as

alvarezsaurians [25]. Conversely, apneumatic sacral vertebrae are present in some theropods, such as the carcharodontosaurid *Tyrannotitan* [46], the therizinosaurid *Nothronychus* [47, 48], and, rarely, in some birds [21].

Caudal pneumaticity has been widely recorded among theropods such as Carcharodontosauridae, Megaraptora, Ornithomimosauria, Therizinosauroidea, Oviraptorosauria and early-diverging alvarezsaurians [17, 33, 34, 48]. Although several clades exhibit caudal pneumaticity, the specimens studied here show that the caudal series of *Bonapartenykus* (though incomplete and represented by disarticulated vertebrae) was pneumatized in both the anterior and middle sections of the tail. This feature is reminiscent of some ornithomimosaurians in which pneumaticity extended to the mid-caudal vertebrae [32]. By contrast, caudal pneumaticity in theropods such as carcharodontosaurids [17], therizinosaurs [49, 50], and abelisaurids is typically limited to the anterior caudals, whereas noasaurids exhibit apneumatic caudal vertebrae [35, 51]. In oviraptorosaurs, caudal pneumatization would have extended to the posterior caudal vertebrae [52, 53, 35]. The evidence collected so far shows that the caudal region of the alvarezsaurids showed extensive development of pneumaticity. Of particular interest is the presence of a caudal vertebra of a parvicursorine from the Hell Creek Formation of the western United States with extensive development of pneumatic structures, and, based on external features, possibly to a greater extent than in South American forms, including *Bonapartenykus* (JGM pers. obs.).

The study of vertebral pneumaticity in Alvarezsauria is limited to external anatomy (such as cortical foramina and natural fractures) and CT-scans performed on *Shishugounykus* [33] and now specimens assigned to *Bonapartenikus*. It appears that the earlier-diverging members of Alvarezsauria, such as *Shishugounykus inexpectus*, exhibit a greater degree of pneumatic invasion than the more derived

forms. However, the absence of cortical foramina in the Asiatic form indicates that the internal pneumatic spaces were likely absent. Thus, here we highlight the first unambiguous evidence of postcranial pneumaticity in an alvarezsaurian theropod based on specimens of *Bonapartenykus*.

It is generally observed that the axial skeleton of alvarezsaurians possesses internal pneumatic spaces, in most cases accompanied by cortical foramina. In addition, *Bannykus*, *Xiyunykus,* and alvarezsaurids share the presence of a foramen that develops above the neural canal in caudal vertebrae. CT-images show that, at least in *Bonapartenikus*, this foramen opens into the neural arch, a feature possibly shared by the other taxa. The data collected from alvarezsaurids do not suggest a linear evolutionary trajectory, but rather points to degree of random variability in the pneumatic features of the axial skeleton (Fig. 8). Future studies, particularly CT-based analyses of additional alvarezsaurid specimens, will be essential to fully understand the extent of pneumatic invasion among members of this clade.

Figure 8. Mapped pneumatic features in the studies by Meso et al. [14]. The studied features are as follows: lateral surface of cervical centra (Ch 1; 0, simple depression; 1, foramina); internal anatomy of cervical centra (Ch 2; 0, absent; 1, present); lateral surface of dorsal centra (Ch 3; 0, simple depression; 1, foramina); internal anatomy of dorsal centra (Ch 4; 0, absent; 1, present); lateral surface of sacral centra (Ch 5; 0, simple depression; 1, foramina); internal anatomy of sacral centra (Ch 6; 0, absent; 1, present); lateral surface of caudal centra (Ch 7; 0, simple depression; 1, foramina); internal anatomy of caudal centra (Ch 8; 0, absent; 1, present); foramen developing above the neural canal in caudal vertebrae (Ch 9; 0, absent; 1, present).

The specimens referred to *Bonapartenykus* examined show a wide range of sizes and ontogenetic stages [14]. For instance, the cervical neural arch (MPCN-Pv 738.28) has irregular neurocentral sutures. This rough aspect of the cervical neural arch base suggests unfused nature with its vertebral centrum and is interpreted as a sign of immaturity. Additionally, the degree of pneumatic invasion within the vertebrae varies considerably between different specimens (possibly linked to ontogenetic stages), particularly in regions such as the tail.

The lack of symmetry is consistent with the hypothesis proposed by Witmer [54] that pneumatization may have been largely opportunistic, potentially related to the emergence of pneumatic diverticula associated with blood vessels, as suggested by Taylor and Wedel [24]. The random or chaotic internal pneumatization is reminiscent of the internal structure of some saurischians, such as a rebbachisaurid caudal vertebra described by Windholz et al. [55]. The pneumaticity is variable because pneumatic diverticula followed blood vessels as they developed, and the course of blood vessels is inherently variable.

The occurrence of pneumatic structures in cervical and anterior-most dorsal vertebrae supports the presence of cervical pneumatic diverticula in *Bonapartenykus*. Furtemore, pneumatic chambers in the neural arch of the posterior cervical vertebra (MPCN-Pv 738.26) connect to the neural canal, which supports the presence of paramedullary diverticula as in birds. Similarly, sacral and caudal pneumaticity indicate the presence of abdominal pneumatic diverticula. Unfortunately, it is not possible to elucidate the presence of other pneumatic features because they do not leave osteological correlates (e.g., posterior thoracic air sacs) or in elements not preserved in the specimens studied by us (e.g., sternum, sternal ribs) [25]. Finally, while reduced bone mass is advantageous for flight in modern birds, its adaptive significance in non-avian dinosaurs, including theropods of varying sizes, remains uncertain [32]. This underscores the need for further studies in the different lineages of saurischians.

Conclusions

This paper presents the first data on the pneumatic features of an alvarezsaurid theropod, in this case *Bonapartenykus ultimus*. The axial skeleton of this species, and possibly other alvarezsaurids, was pneumatized. This is supported by the presence of cortical openings communicating with internal air spaces in the vertebral elements. The axial skeleton of *B. ultimus* was apparently pneumatized from the cervical back to the mid-tail region. Members of Alvarezsauridae show random variability in the pneumatic structures of the axial series. This study emphasizes the need for studies of the pneumatic system, examining both the external and internal anatomy of the specimens. 6

Acknowledgements

We want to thank P. Chafrat from the Museo Patagónico de Ciencias Naturales, General Roca, Río Negro Province, Argentina. The authors gratefully acknowledge support from the "Fundacion Patagonica de Ciencias Naturales" and "Sanatorio Juan XXIII", who made the CT-scanning posible. MP is supported by the School of Life Sciences of The Chinese University of Hong Kong.

References

1. Bonaparte JF. Los vertebrados fósiles de la Formación Río Colorado, de la Ciudad de Neuquén y cercanías, Cretácico Superior, Argentina. Revista del Museo Argentino de Ciencias Naturales. 1991; 4: 17–123.
2. Perle A, Chiappe LM. Barsbold R. Skeletal morphology of *Mononykus olecranus* (Theropoda: Avialae) from the late Cretaceous of Mongolia. American Museum Novitates, 1994; 3105: 1–29.
3. Novas FE. Alvarezsauridae, Cretaceous basal birds from Patagonia and Mongolia. Memoirsqueensland Museum, 1996; 39: 675–702.
4. Martinelli AG, Vera EI. *Achillesaurus manazzonei*, a new alvarezsaurid theropod (Dinosauria) from the Late Cretaceous Bajo de la Carpa Formation, Río Negro Province, Argentina. Zootaxa, 2007; 1582: 1–17.
5. Longrich NR, Currie PJ. *Albertonykus borealis*, a new alvarezsaur (Dinosauria: Theropoda) from the Early Maastrichtian of Alberta, Canada: Implications for the

systematics and ecology of the Alvarezsauridae. Cretaceous Research, 2009; 30: 239–252.

6. Agnolín FL, Powell JE, Novas FE, Kundrát M. New alvarezsaurid (Dinosauria, Theropoda) from uppermost Cretaceous of north-western Patagonia with associated eggs. Cretaceous Research, 2012; 35: 33–56.
7. Makovicky PJ, Apesteguía S, Gianechini FA. A new coelurosaurian theropod from the Lan Buitrera fossil locality of Río Negro, Argentina. Fieldiana Life and Earth Sciences, 2012; 2012: 90–98.
8. Pittman M, Xu X, Stiegler JB. The taxonomy of a new parvicursorine alvarezsauroid specimen IVPP V20341 (Dinosauria: Theropoda) from the Upper Cretaceous Wulansuhai Formation of Bayan Mandahu, Inner Mongolia, China. PeerJ, 2015; 3: e986.
9. Averianov A, Sues HD. The oldest record of Alvarezsauridae (Dinosauria: Theropoda) in the Northern Hemisphere. Plos one, 2017; 12: e0186254.
10. Fowler DW, Wilson JP, Fowler EA, Noto CR, Anduza D, Horner JR. *Trierarchuncus prairiensis* gen. et sp. nov., the last alvarezsaurid: Hell Creek Formation (uppermost Maastrichtian), Montana. Cretaceous Research, 2020; 116: 104560.
11. Qin Z, Zhao Q, Choiniere JN, Clark JM, Benton MJ, Xu X. Growth and miniaturization among alvarezsauroid dinosaurs. Current Biology, 2021; 31: 3687–3693.
12. Makovicky PJ, Apesteguia S, Gianechini FA. A new, almost complete specimen of *Alnashetri cerropoliciensis* (Dinosauria: Theropoda) impacts our understanding of alvarezsauroid evolution. Ameghiniana, 2016; 53: 6.

13. Novas FE. Anatomy of *Patagonykus puertai* (Theropoda, Avialae, Alvarezsauridae), from the Late Cretaceous of Patagonia. Journal of Vertebrate Paleontology, 1997; 17: 137–166.
14. Meso JG, Pol D, Chiappe L, Qin Z, Diaz-Martínez I, Gianechini F, Apesteguia S, Makovycky PJ, Pittman M. Body size and evolutionary rate analyses reveal complex evolutionary history of Alvarezsauria. Cladistics. In press.
15. Salgado L, Coria RA, Arcucci AB, Chiappe LM. Restos de Alvarezsauridae (Theropoda, Coelurosauria) en la Formación Alien (Campaniano-Maastrichtiano), en Salitral Ojo de Agua, Provincia de Río Negro, Argentina. Andean geology, 2009; 36: 67–80.
16. Meso JG, Choiniere J, Baiano MA, Brusatte SL, Canale JI, Salgado L, Pol D, Pittman M. New information on *Bonapartenykus* (middle Campanian-lower Mastrichtian) of Rio Negro Province, Patagonia, Argentina clarifies the Patagonykinae body plan. ~~Plos One~~. In press.
17. Britt BB. Pneumatic postcranial bones in dinosaurs and other archosaurs. Doctoral thesis, University of Calgary, 1993; 4580–4580.
18. Britt BB. Postcranial pneumaticity. In: Currie PJ, Padian K, editors. The encyclopedia of dinosaurs. Oxford, UK: Academic Press, 1997; 590–593.
19. Wedel MJ. Vertebral pneumaticity, air sacs, and the physiology of sauropod dinosaurs. Paleobiology, 2003; 29: 243–255.
20. Wedel MJ. Postcranial skeletal pneumaticity in sauropods and its implications for mass estimates. In: Wilson JA, Curry-Rogers K, editors. The sauropods: evolution and paleobiology. Berkeley: University of California Press, 2005; 201–228.

21. Wedel MJ. Evidence for bird-like air sacs in saurischian dinosaurs. Journal of Experimental Zoology, 2009; 311A: 611e628.

22. O'Connor PM. Postcranial pneumaticity: an evaluation of soft-tissue influences on the postcranial skeleton and the reconstruction of pulmonary anatomy in archosaurs. Journal of Morphology, 2006; 267: 1199–1226.

23. O'Connor PM. Evolution of archosaurian body plans: skeletal adaptations of an air-sac-based breathing apparatus in birds and other archosaurs. Journal of Experimental Zoology. Part A, Ecological Genetics and Physiology, 2009; 311: 629–646.

24. Taylor M, Wedel M. Why is vertebral pneumaticity in sauropod dinosaurs so variable? Qeios, 2021; 4: 1–13.

25. O'Connor PM, Claessens LP. Basic avian pulmonary design and flowthrough ventilation in non-avian theropod dinosaurs. Nature, 2005; 253–256.

26. Yates AM, Wedel MJ, Bonnan MF. The early evolution of postcranial skeletal pneumaticity in sauropodomorph dinosaurs. Acta Palaeontologica Polonica, 2012; 57: 85–100.

27. Windholz GJ, Coria RA, Zurriaguz VL. Vertebral pneumatic structures in the Early Cretaceous sauropod dinosaur *Pilmatueia faundezi* from northwestern Patagonia, Argentina. Lethaia, 2019; 53: 369–381.

28. Gianechini FA, Zurriaguz VL. Vertebral pneumaticity of the paravian theropod *Unenlagia comahuensis*, from the Upper Cretaceous of Patagonia, Argentina. Cretaceous Research, 2021; 127: 104925.

29. Cerda IA, Salgado L, Powell JE. Extreme postcranial pneumaticity in sauropod dinosaurs from South America. Paläontologische Zeitschrift, 2012; 86: 441–449.

30. Windholz GJ, Carballido JL, Coria RA, Zurriaguz VL, Rauhut OW. How pneumatic were the presacral vertebrae of dicraeosaurid (Sauropoda: Diplodocoidea) dinosaurs? Biological Journal of the Linnean Society, 2022; 138: 103–120.
31. Benson RB, Butler RJ, Carrano M,T, O'Connor PM. Air-filled postcranial bones in theropod dinosaurs: physiological implications and the ‘reptile’–bird transition. Biological Reviews, 2012; 87: 168–193.
32. Watanabe A, Leone Gold ME, Brusatte SL, Benson RB, Choiniere J, Davidson A, Norell MA. Vertebral pneumaticity in the ornithomimosaur *Archaeornithomimus* (Dinosauria: Theropoda) revealed by computed tomography imaging and reappraisal of axial pneumaticity in Ornithomimosauria. PloS one, 2015; 10(12), e0145168.
33. Qin Z, Clark J, Choiniere J, Xu X. A new alvarezsaurian theropod from the Upper Jurassic Shishugou Formation of western China. Scientific Reports, 2019; 9: 11727.
34. Aranciaga Rolando M, Garcia Marsa J, Novas F. Histology and pneumaticity of *Aoniraptor libertatem* (Dinosauria, Theropoda), an enigmatic mid-sized megaraptoran from Patagonia. Journal of Anatomy, 2020; 237: 741–756.
35. Baiano MA, Coria RA, Chiappe LM, Zurriaguz V, Coria L. Osteology of the axial skeleton of *Aucasaurus garridoi*: phylogenetic and paleobiological inferences. PeerJ, 2023; 11: e16236.
36. Wilson JA. A nomenclature for vertebral laminae in sauropods and other saurischian dinosaurs. Journal of vertebrate Paleontology, 1999; 19: 639–653.

37. Wilson JA. New vertebral laminae and patterns of serial variation in vertebral laminae of sauropod dinosaurs. Contributions from the Museum of Paleontology, University of Michigan, 2012; 32: 91–110.

38. Wilson JA, D'Emic MD, Ikejiri T, Moacdieh EM, Whitlock JA. A nomenclature for vertebral fossae in sauropods and other saurischian dinosaurs. PLoS One, 2011; 6: e17114.

39. Chiappe LM, Norell MA, Clark JM. The Cretaceous, short-armed Alvarezsauridae: *Mononykus* and its kin. Mesozoic birds: above the heads of dinosaurs, 2002; 87–120.

40. Senter P. Function in the stunted forelimbs of *Mononykus olecranus* (Theropoda), a dinosaurian anteater. Paleobiology, 2005; 31: 373–381.

41. Choiniere JN, Clark JM, Norell MA, Xu X. Cranial osteology of *Haplocheirus sollers* Choiniere et al., 2010 (Theropoda: Alvarezsauroidea). American Museum Novitates, 2014; 2014: 1–44.

42. Choiniere JN, Neenan JM, Schmitz L, Ford DP, Chapelle KE, Balanoff AM, Simpla JS, Georgi JA, Walsh SA, Norell MA, Xu X, Clark JM, Benson RB. Evolution of vision and hearing modalities in theropod dinosaurs. Science, 2021; 372: 610–613.

43. Meso JG, Qin Z, Pittman M, Canale JI, Salgado L, Díaz VD. Tail anatomy of the Alvarezsauria (Theropoda, Coelurosauria), and its functional and behavioural implications. Cretaceous Research, 2021; 124: 104830.

44. Qin Z, Liao CC, Benton MJ, Rayfield EJ. Functional space analyses reveal the function and evolution of the most bizarre theropod manual unguals. Communications Biology, 2023; 6; 1–181.

45. Forster CA, O'connor PM, Chiappe LM, Turner AH. The osteology of the Late Cretaceous paravian *Rahonavis ostromi* from Madagascar. Palaeontologia Electronica, 2020; 23:a29.
46. Novas FE, de Valais S, Vickers-Rich P, Rich T. A large Cretaceous theropod from Patagonia, Argentina, and the evolution of carcharodontosaurids. Naturwissenschaften, 2005; 92: 226–230.
47. Kirkland JI, Wolfe DG. First definitive therizinosaurid (Dinosauria; Theropoda) from North America. Journal of Vertebrate Paleontology, 2001; 21: 410–414.
48. Smith DK, Sanders RK, Wolfe DG. Vertebral pneumaticity of the North American therizinosaur *Nothronychus*. Journal of Anatomy, 2021; 238: 598–614.
49. Zanno LE, Gillette DD, Albright LB, Titus AL. A new North American therizinosaurid and the role of herbivory in 'predatory'dinosaur evolution. Proceedings of the Royal Society B: Biological Sciences, 2009; 276: 3505–3511.
50. Zanno LE. Osteology of *Falcarius utahensis* (Dinosauria: Theropoda): characterizing the anatomy of basal therizinosaurs. Zoological Journal of the Linnean Society, 2010; 158: 196–230.
51. Carrano MT, Loewen MA, Sertich JJ. New materials of *Masiakasaurus knopfleri* Sampson, Carrano, and Forster, 2001, and implications for the morphology of the Noasauridae (Theropoda: Ceratosauria). Smithsonian Contributions to Paleobiology, 2011; 95: 1–53.
52. Xu X, Tan Q, Wang J, Zhao X, Tan L. A gigantic bird-like dinosaur from the Late Cretaceous of China. Nature, 2007; 447: 844–847.

53. Balanoff AM, Norell MA. Osteology of *Khaan mckennai* (Oviraptorosauria: Theropoda). Bulletin of the American Museum of Natural History, 2012; 2012: 1–77.

54. Witmer LM. The evolution of the antorbital cavity of archosaurs: a study in soft-tissue reconstruction in the fossil record with an analysis of the function of pneumaticity. Journal of Vertebrate Paleontology, 1997; 17: 1–76.

55. Windholz GJ, Porfiri JD, Dos Santos D, Bellardini F, Wedel M.J. A well-preserved vertebra provides new insights into rebbachisaurid sauropod caudal anatomical and pneumatic features. Acta Palaeontologica Polonica, 2024; 69: 39–47.

Figure 1

Click here to access/download;Figure;Figure 1.tif

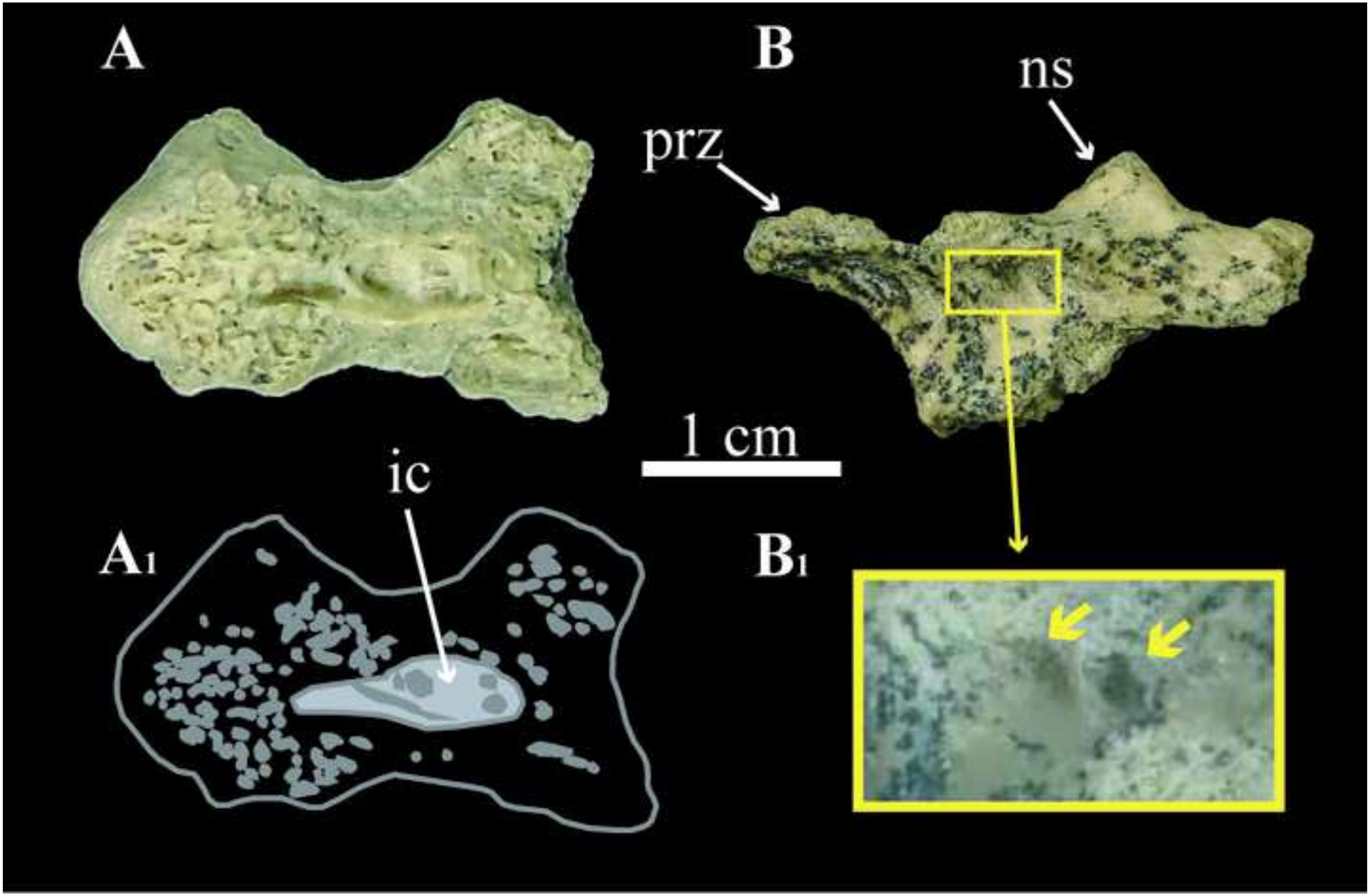

Figure 2

Click here to access/download;Figure;Figure 2.tif

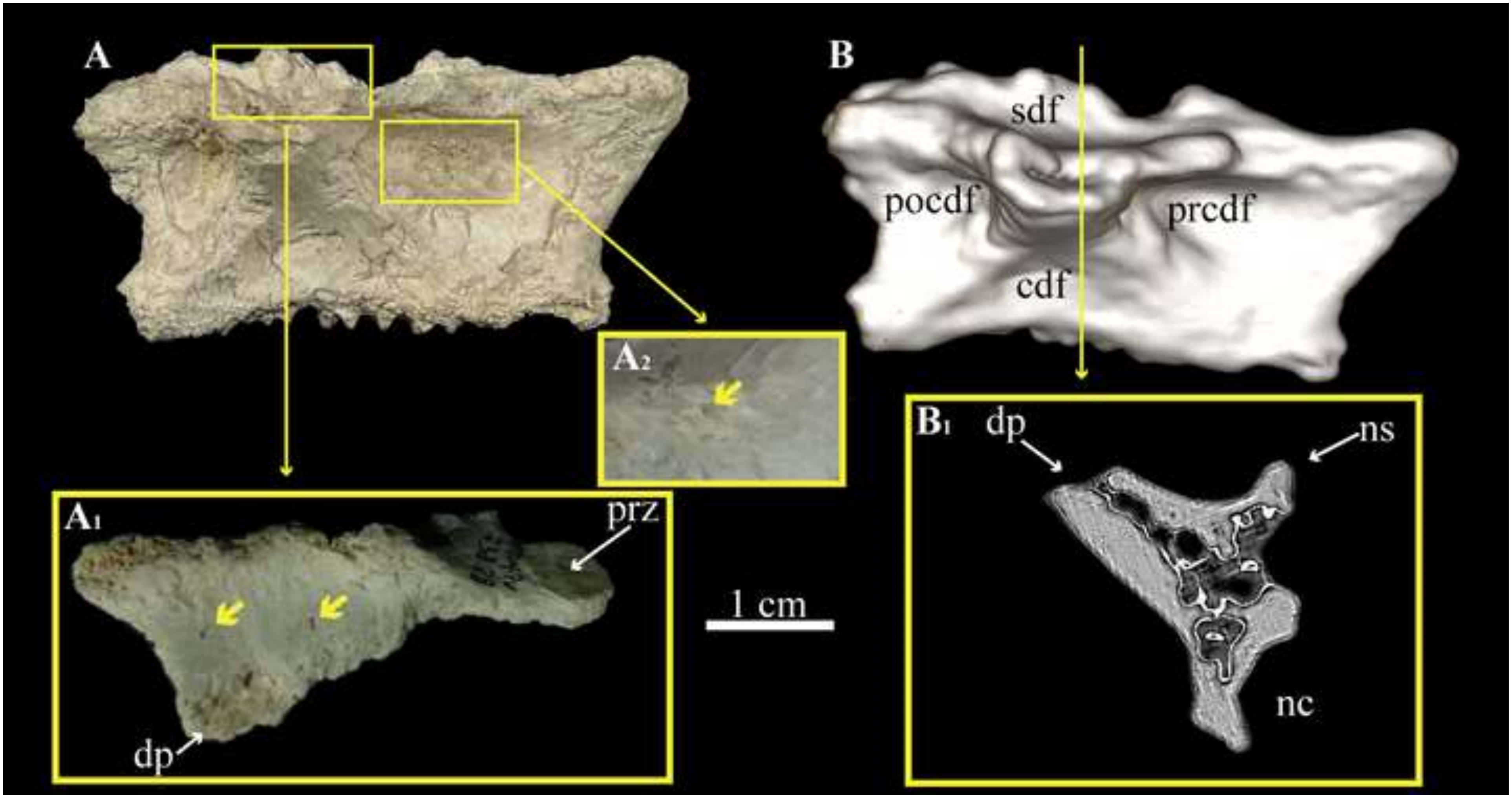

Figure 3

Click here to access/download;Figure;Figure 3.tif

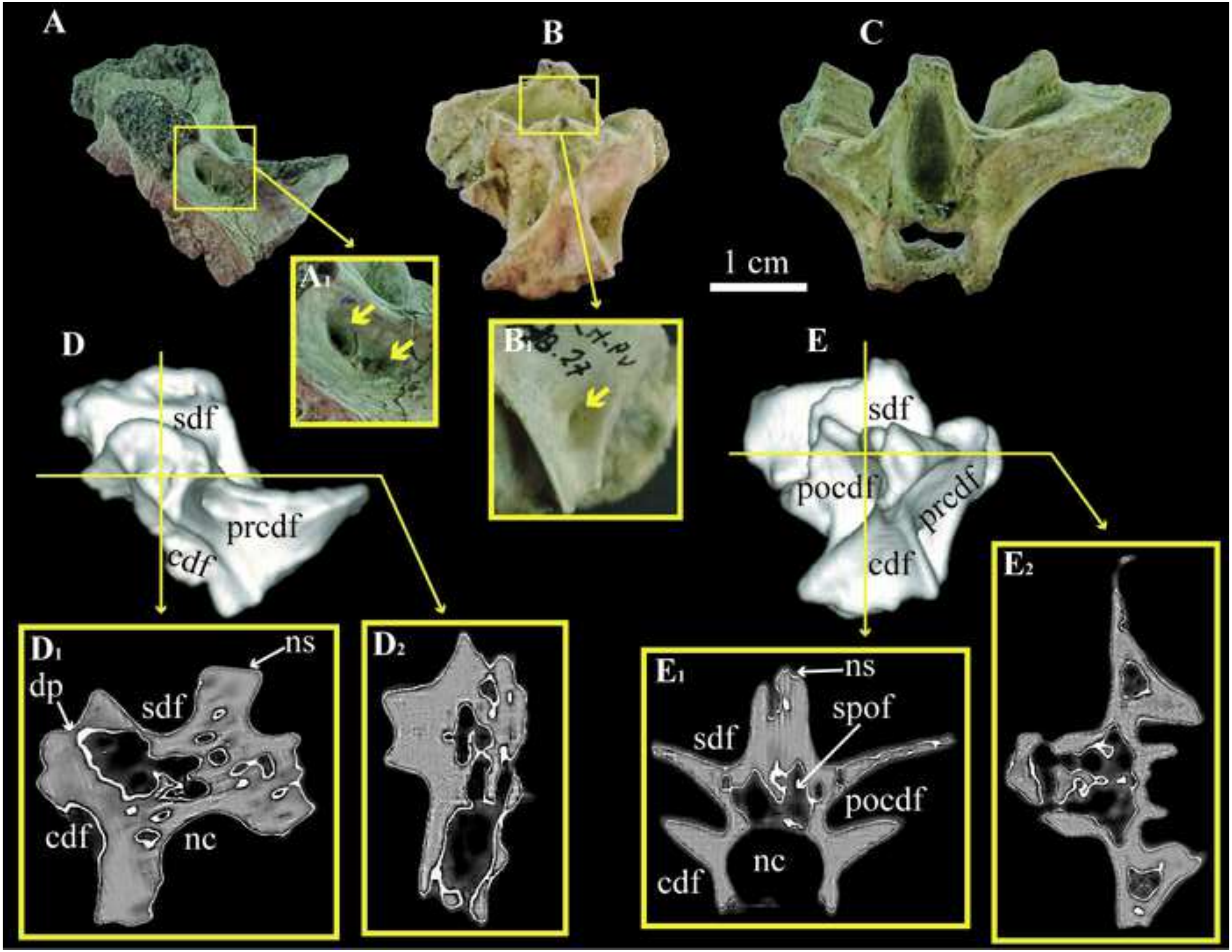

Figure 4

Click here to access/download;Figure;Figure 4.tif

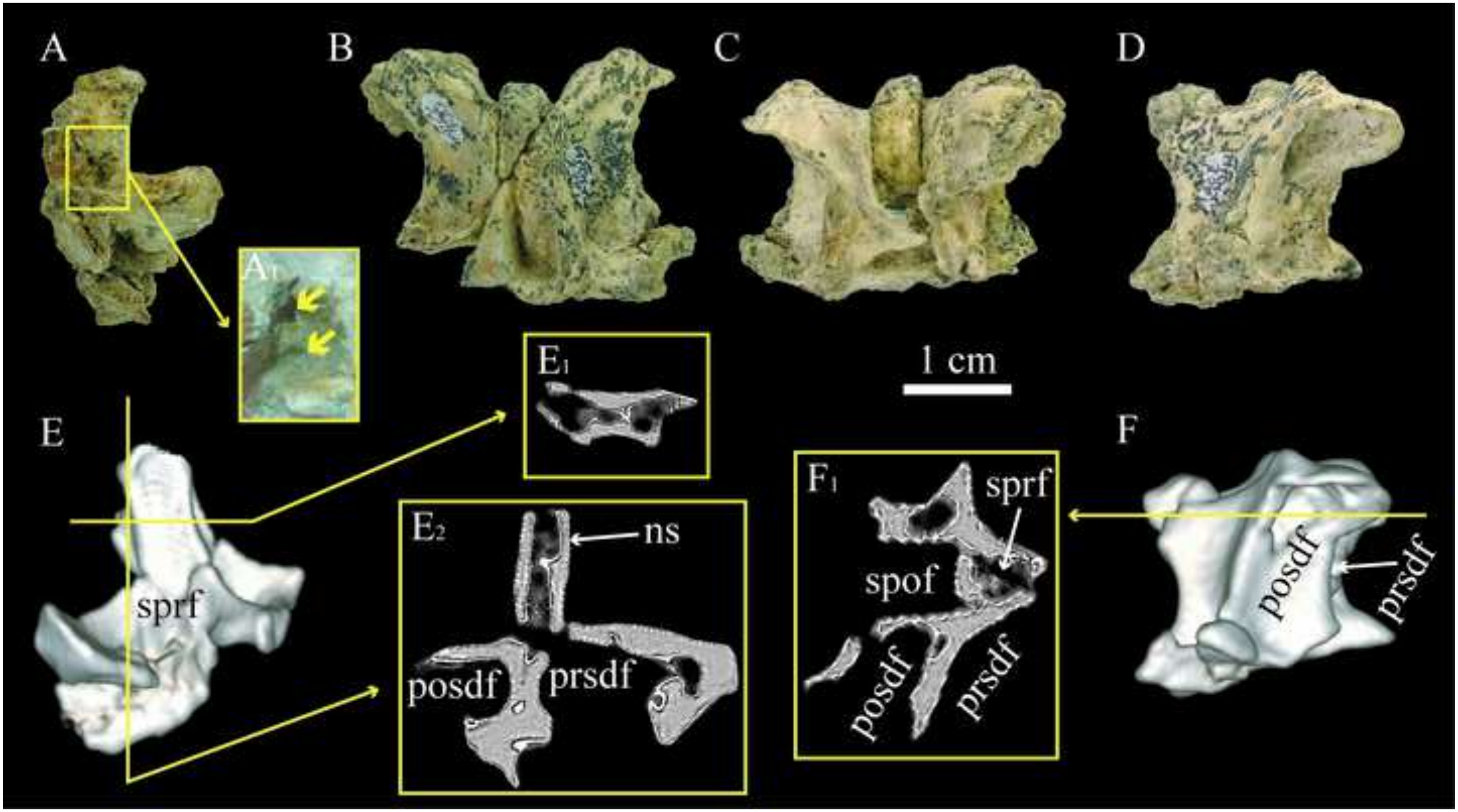

Figure 5

Click here to access/download;Figure;Figure 5.tif

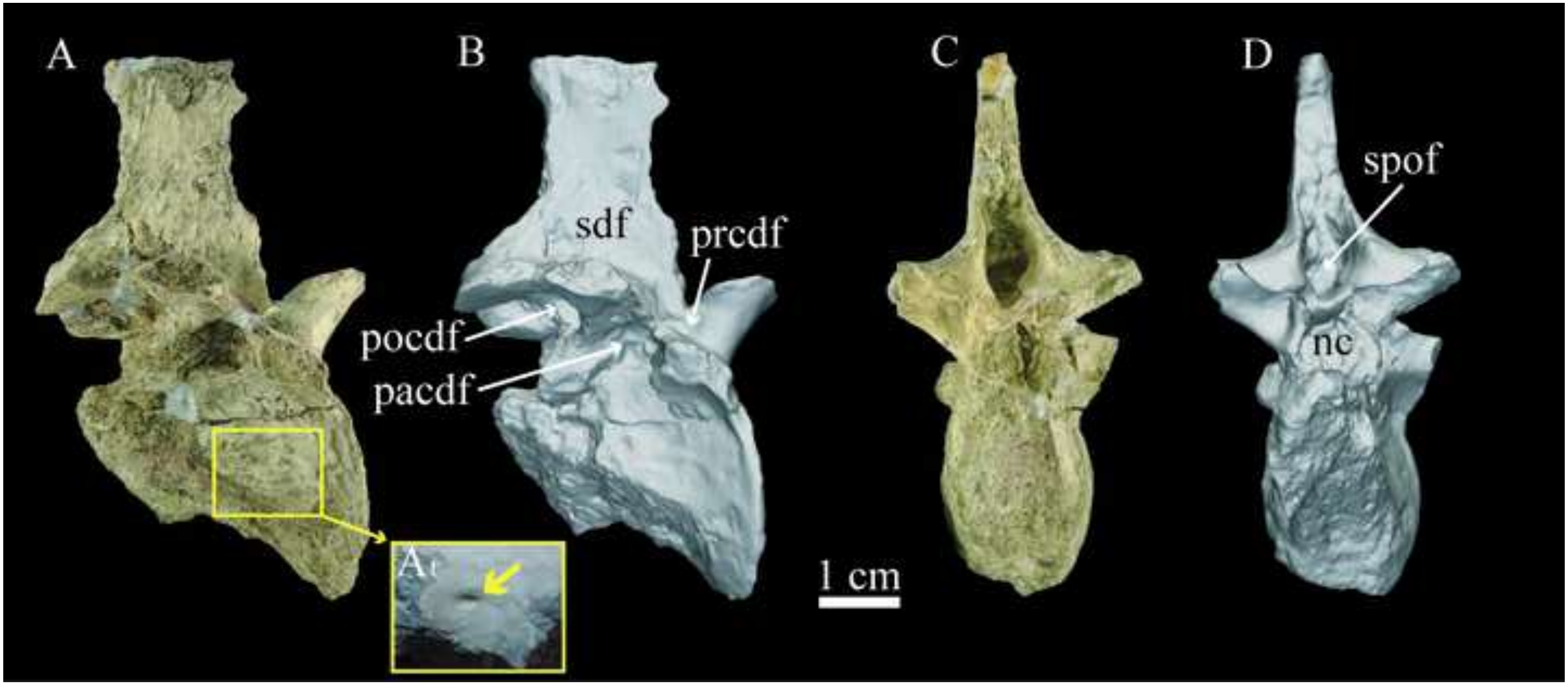

Figure 5

Click here to access/download;Figure;Figure 6.tif

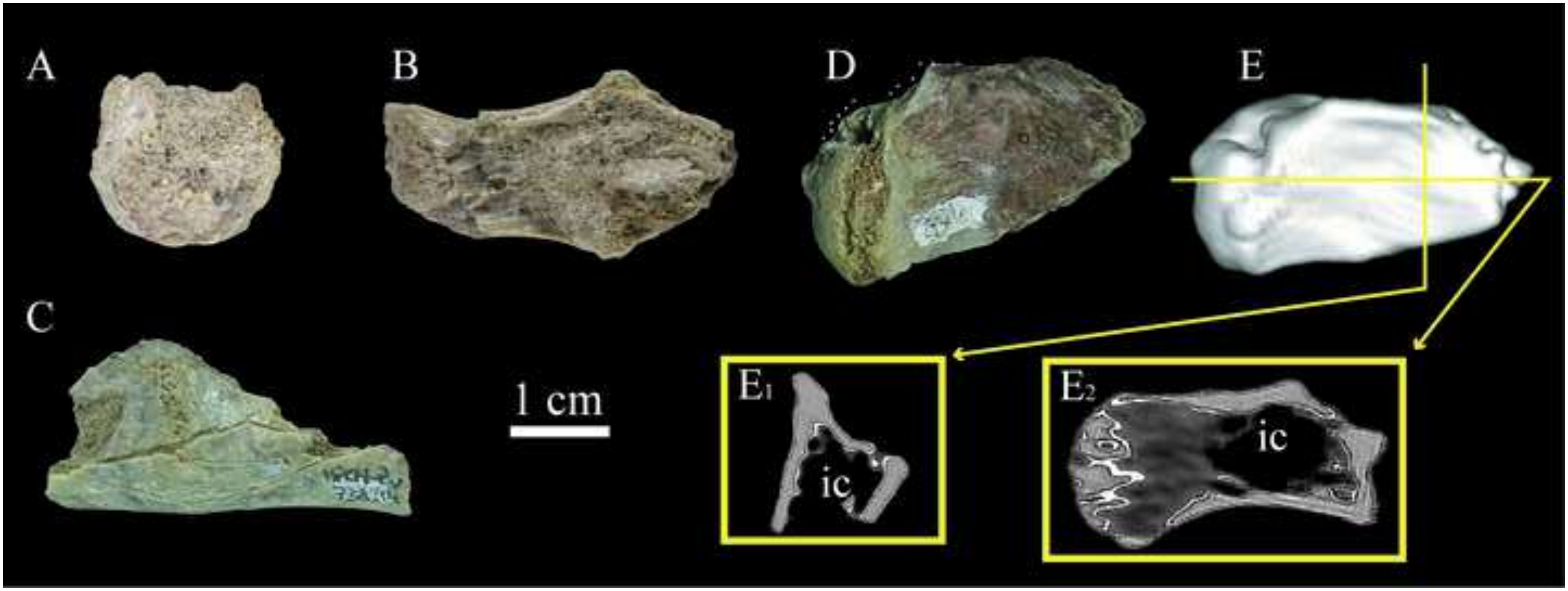

Figure 7

Click here to access/download;Figure;Figure 7.tif

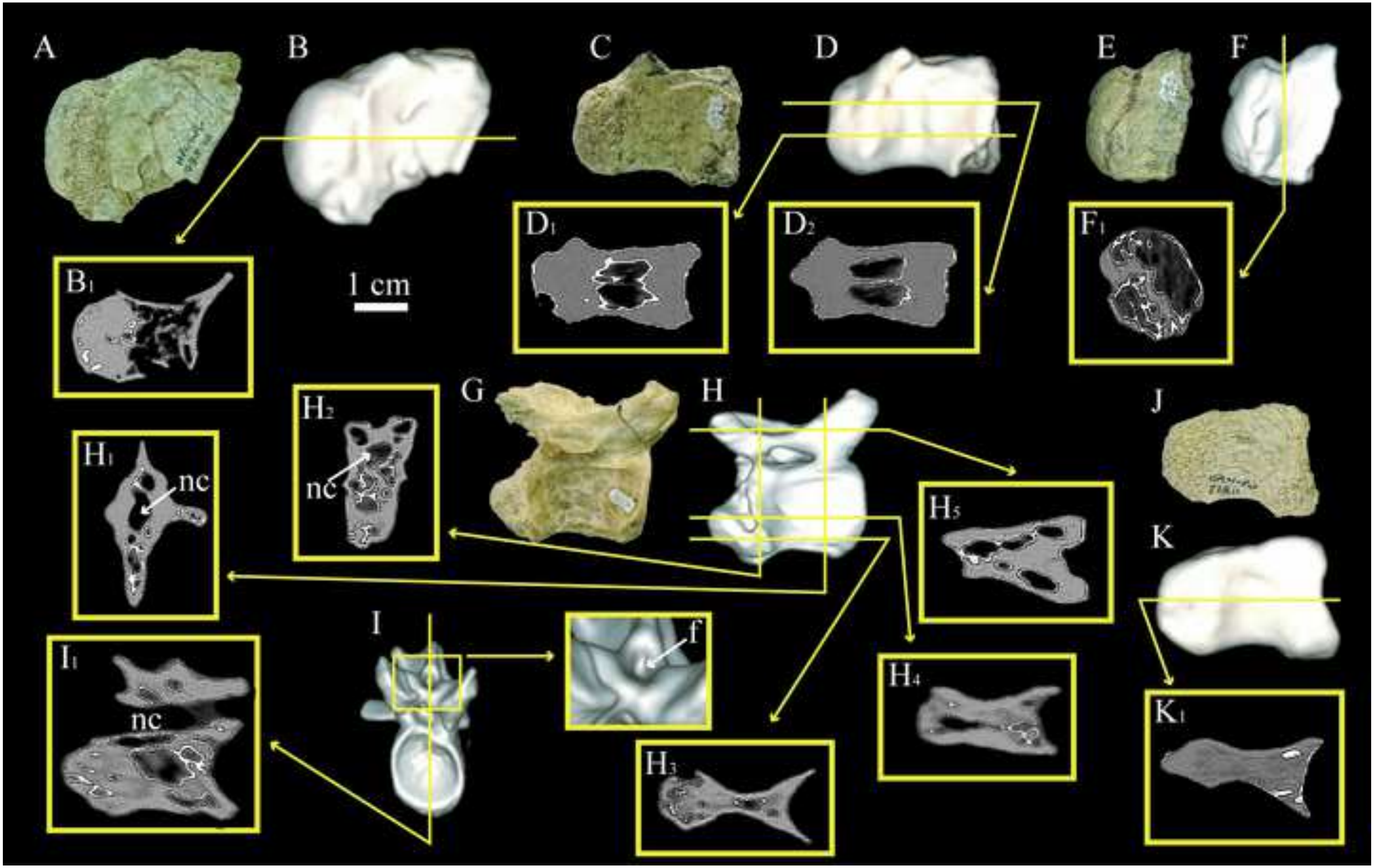

Figure 8 Click here to access/download;Figure;Figure 8.tif

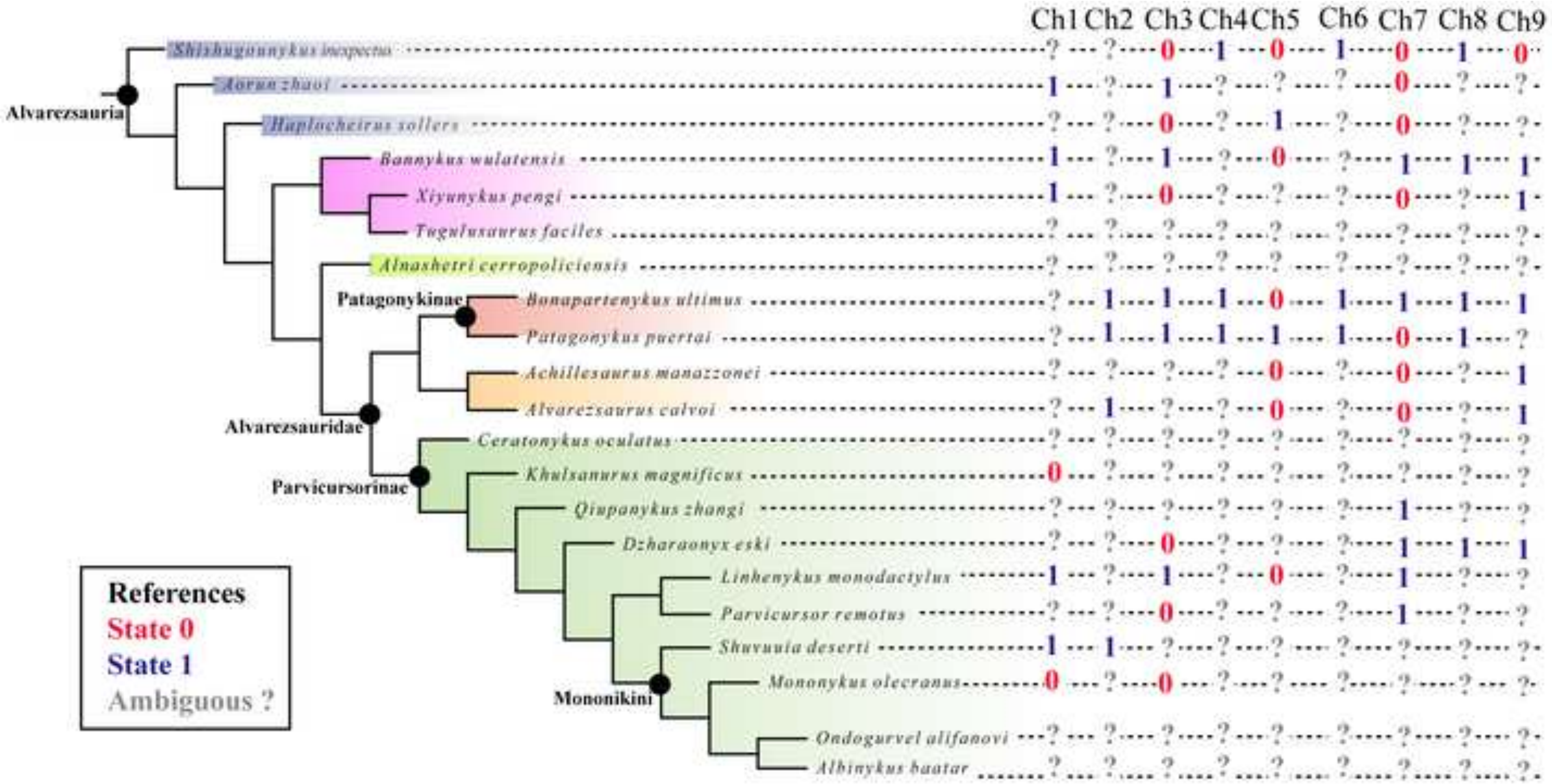

Supplement: Attachment — Submitted filename: PONE-D-24-50106_REVIEW.pdf [file pone.0320121.s002.pdf]
